# Salidroside: An Overview of Its Promising Potential and Diverse Applications

**DOI:** 10.3390/ph17121703

**Published:** 2024-12-17

**Authors:** Keke Liang, Shuhe Ma, Kai Luo, Renjie Wang, Chenrong Xiao, Xianxie Zhang, Yue Gao, Maoxing Li

**Affiliations:** 1 College of Pharmacy, Gansu University of Chinese Medicine, Lanzhou 730000, China; 18394248206@163.com (K.L.); mclsxka@163.com (S.M.); 18294623280@163.com (K.L.); wangrenjiegy@163.com (R.W.); 2 Department of Pharmaceutical Sciences, Beijing Institute of Radiation Medicine, Beijing 100850, China; xiaocr@sina.com (C.X.); zhangxianxie@163.com (X.Z.); 3 National Key Laboratory of Kidney Diseases, Beijing 100850, China

**Keywords:** salidroside, pharmacology, signaling pathways, clinical application, safety, review

## Abstract

Salidroside, a phenolic compound isolated from various *Rhodiola* plants, is the principal active constituent of Traditional Chinese Medicine known for its adaptogenic properties. Due to the challenging environment of *Rhodiola* species, such as high altitude, high radiation, drought, and hypoxia, the source of salidroside is scarce. However, numerous studies have shown that salidroside has a range of biological activities, including cardiovascular and central nervous system activity, and anti-hypoxia, anti-inflammatory, and anti-aging activities. Although previous studies have partially summarized the pharmacological effects of salidroside, the overall pharmacological effects have not been analyzed. Hence, this review will systematically summarize the isolation, purification, synthesis, derivatization, pharmacological activity, pharmacokinetics, clinical application, and safety of salidroside. It is expected to provide new insights for the further research and pharmaceutical development of salidroside.

## 1. Introduction

**Figure 1:**
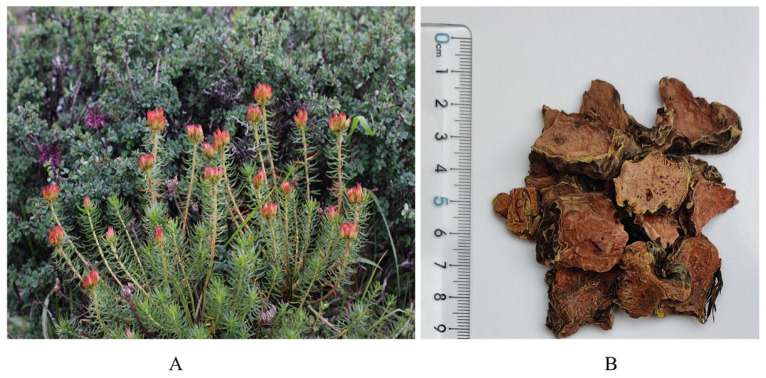
Rhodiola plants and herbal slices. (**A**) Rhodiola plants. (**B**) Rhodiola herbal slices.

**Figure 2:**
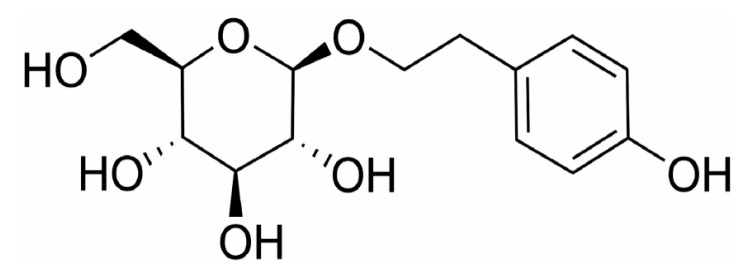
The structure of salidroside.

*Rhodiola* is a perennial flowering herb, which grows mainly in the Himalayas, Northwest Asia, North America, and other alpine regions (Figure 1) [1]. It is known as ‘plateau *Panax ginseng*’ for its tenacious vitality and resistance to hypoxic conditions [2]. Salidroside is a phenolic compound widely found and extracted from the rhizomes and tubers of *Rhodiola* plants (Figure 2). Compared with other adaptogens in traditional medicine, salidroside has unique value in anti-hypoxia, antioxidation, and anti-inflammation. For example, Astragaloside and Ginsenoside indirectly play an anti-hypoxia role by enhancing the body’s immune function and improving cardiac function. Salidroside can directly stimulate the production of erythropoietin, thereby improving the oxygen-carrying capacity of blood and effectively alleviating hypoxia damage. However, the content of natural salidroside in Rhodiola is only 0.5~0.8%, the direct extraction process is complicated, and the extraction rate is low [3,4]. Therefore, producing salidroside by chemical synthesis and biotechnology has become a research hotspot. At the same time, salidroside has been proven as an environmentally adaptable compound with anti-hypoxia, anti-inflammatory, anti-tumor, and neuroprotective activities. It has been used and exhibits an important application value in the military, aerospace, and healthcare medicines [5].

Therefore, a large number of studies on salidroside have been carried out and the relevant literature has been reported. For example, Jin et al. [6] reviewed the protective effect of salidroside on the central nervous system; Wang et al. [7] summarized the anti-tumor activity and synthetic pathway of salidroside. However, the current research only summarizes the biological activity of salidroside from a single aspect. A comprehensive review that provides a complete profile of the isolation, purification, synthesis, analogs, pharmacology, pharmacokinetics, and safety of salidroside is still lacking. In the present study, with the help of some scientific search engines and databases, including Google Scholar, Web of Science, Pubmed, and Chinese National Knowledge Infrastructure (CNKI), we summarize the recent research progress of the above aspects. We hope that this review will highlight some present issues and future perspectives and help us to develop and utilize salidroside more efficiently and safely.

## 2. Distribution of *Rhodiola* Plants

**Figure 3:**
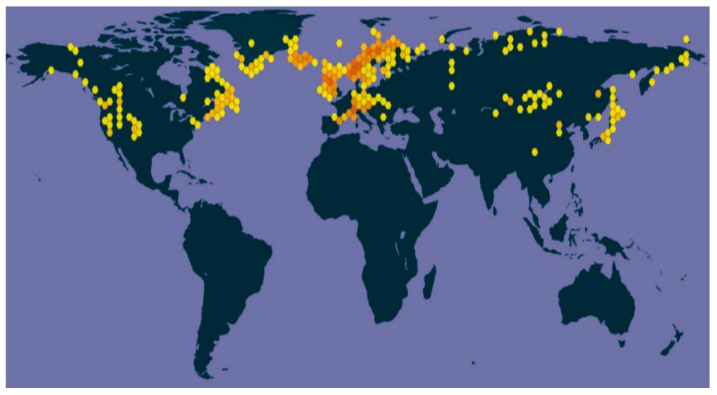
The distribution of Rhodiola (the color of the hexagons represents the number of distribution records in each hexagon) [11].

*Rhodiola*, a perennial herb or subshrub in the Crassulaceae family, has a slow natural regeneration cycle of 7–8 years. With over 90 species globally, most Rhodiola species thrive in alpine regions of the Northern Hemisphere. These plants typically grow in challenging environments such as limestone or granite mountain glaciers, mountain grasslands, or rocky valleys at elevations ranging from 3500 to 5000 m. Some species can also be found at lower altitudes, around 2000 m, in alpine grasslands, vegetation, or ditches near rocky areas. Notably, certain Rhodiola species exist at extreme elevations of 5000 m in regions like North America, East Asia, Central Asia, and Siberia (Figure 3) [8].

There are 73 species, 2 subspecies, and 7 varieties in China, accounting for about 85% of the world’s Rhodiola resources, which are mainly distributed in northeast, north, northwest, and southwest regions. The Qinghai–Tibet Plateau has a special geographical location, with a distribution of 55 species. The dominant one is Rhodiola rosea, which has been collected in the “Standard of Tibetan Medicine of the Ministry of Health of the People’s Republic of China” and “Chinese Pharmacopoeia”. In addition, Rhodiola sachalinensis has been artificially cultivated in Yanbian, Jilin Province, which is expected to alleviate the scarcity of Rhodiola resources [9,10].

## 3. Content of Salidroside from *Rhodiola*

HPLC is commonly used for the determination of salidroside. The content of salidroside (C_14_H_20_O_7_) stipulated in the 2020 edition of the Chinese Pharmacopoeia should not be less than 0.50%. At present, *Rhodiola sachalinensisn* A. Bor or *Rhodiola crenulata* H. Ohba is commonly used in China. *Rhodiola rosea* is mainly used in the United States and Europe [12]. According to statistics, there are a total of more than 90 species of *Rhodiola* in the world, and 73 species, 2 subspecies, and 7 varieties in China. Therefore, the content of the salidroside of different species is different. For example, some scholars have measured the content of salidroside in six kinds of *Rhodiola* produced in Qinghai [13], among which the content of salidroside in *R. algida* is rich in 3.13%, while that in *R. juparensis* is only 0.64%, indicating that the content of salidroside is related to the species of *Rhodiola*.

The content of effective components of Traditional Chinese Medicine (TCM) is closely related to the natural environment such as soil, light, and humidity. The factors of origin also have a certain effect on the content of salidroside. For example, the content of salidroside in R. rosea in different distributing areas (Xinjiang, Hebei, Russia) is 0.337%, 0.38%, and 0.48%, respectively. The content of salidroside in *R. crenulata* (HK. f. et Thoms.) H. Ohba in different distributing areas (Jilin, Sichuan, Xizang) is also relatively close, among 1.52~1.12%. It can be seen that the factors of origin also have a certain effect, but the effect is small.

The content of salidroside is also affected by storage time. For example, the contents of salidroside in *R. yunnanensis* and *R. fastigiata* collected in 2002 were 0.30% and 0.016%, respectively, while salidroside could not be detected in the same samples stored more than 3 years with the same methods. The researchers analyzed that this may be due to the partial decomposition of salidroside into aglycones with the extension of storage time [14]. On the other hand, some scholars also determined the content of salidroside in *R. crenulata* (HK. f. et Thoms.) H. Ohba with different storage years, and the content of salidroside was between 0.38% and 1.56% [15,16,17]. Therefore, the storage time should be suitable for the characteristics of the medicinal material.

In addition, the content of active ingredients in different medicinal parts is not the same. Some scholars have systematically tested different parts of *R. crenulata* (HK. f. et Thoms.) H. Ohba. The analysis showed that the content of salidroside was 35.00 mg/g (petal), 18.89 mg/g (root), and 1.59 mg/g (stem) [18,19,20]. Obviously, salidroside not only can be found in the root but also in the aerial part. Since most wild *Rhodiola* is only used with the roots, collecting a large number of roots will aggravate resource depletion and destroy alpine vegetation. The aerial part of *Rhodiola* also has a certain content of salidroside and can be used to extract salidroside in future research [21,22,23,24].

Above all, the content of salidroside in *Rhodiola* may be affected by many factors. The content of salidroside has been used to evaluate the quality of *Rhodiola*. On the other hand, TCM is often composed of several materials and has numerous ingredients with a variety of pharmacology activities. Therefore, the quality of Traditional Chinese Medicine (TCM) cannot be evaluated with one compound. The contents of salidroside are shown in Table 1.

## 4. Isolation and Purification of Salidroside

**Figure 4:**
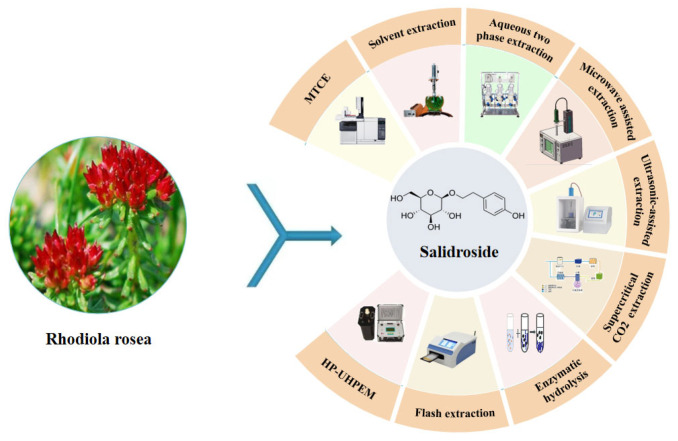
The extraction method of salidroside.

The isolation and purification of salidroside is a key process for measuring the content of salidroside. At present, the main methods used in the isolation and purification of salidroside include solvent extraction, double-water-phase extraction, microwave-assisted extraction, ultrasonic-assisted extraction, supercritical CO_2_ extraction, enzyme hydrolysis, flash extraction, ultra-high-pressure extraction, multi-technology-coupling extraction, and so on (Figure 4).

### 4.1. Solvent Extraction Method (SEM)

The solvent extraction method is a common extraction method with simple operation and low cost. Liu et al. [25] optimized the extraction process of salidroside and found that the optimal solution of the water extraction method was to add eight times the amount of water to the medicinal material and extract it by boiling for three times, each time for 2 h. At this time, the extraction rate of salidroside was up to 1.0339%. On the other hand, some scholars optimized the extraction process of salidroside by a single-factor analysis. The extraction rate of salidroside obtained by alcohol extraction was 5.72% [26]. Although the solvent extraction method is simple to operate, if the extraction conditions are not strictly controlled, such as temperature, time, etc., it may also cause the oxidation of salidroside or other chemical reactions, affecting its stability and thus reducing its biological activity.

### 4.2. Aqueous Two-Phase Extraction

Aqueous two-phase extraction, also known as the aqueous two-phase distribution method, is a new liquid–liquid extraction technology, with mild conditions, simple equipment operation, and easy to large-scale production. Guo [27] found in the two-phase aqueous extraction of salidroside, when the two-phase aqueous system was 20% PEG1000, 20% [NH_4_]_2_SO_4_, and 1% KCl, the extraction rate was 95.32%. Under certain conditions, the extraction rate of salidroside by the two-phase water extraction method was higher (such as 95.32% under a specific system ratio). In practice, however, it is difficult to accurately maintain the ratio of the two-phase water systems such as 20% PEG1000, 20% [NH_4_]_2_SO_4_, and 1% KCl. The ratio deviation causes the extraction rate to be unstable. In addition, the two-phase water extraction system is complex, and the phase conversion and distribution process exposes salidroside to a variable environment, and the local concentration and pressure changes during phase separation increase the risk of chemical reactions or physical changes, affecting its stability.

### 4.3. Ultrasonic-Assisted Extraction Method

The ultrasonic-assisted extraction method mainly makes use of the strong vibration, cavitation effect, and thermal effect produced by ultrasonic waves in the liquid solution, which can improve the extraction efficiency, shorten the extraction time, and save the solvent. Dong et al. [28] optimized the ultrasonic extraction process of salidroside through single-factor and orthogonal experiments. It was found that the optimal process parameters were a 60% ethanol concentration, liquid–solid ratio of 20:1 (mL:g), extraction temperature of 40 °C, and extraction time of 75 min. The influence of the factors on the extraction rate was the temperature > liquid–solid ratio > extraction time > ethanol concentration. Furthermore, Gao et al. [29] also studied the process of the ultrasound-assisted extraction of salidroside and obtained the extraction rate of salidroside at 1.638%. However, in practice, the strong vibration, cavitation effect, and thermal effect produced by ultrasonic waves may damage the chemical structure of salidroside. For example, excessive vibration and thermal effects may cause chemical bonds in salidroside molecules to break, rendering them inactive. In the process of large-scale extraction, the accumulation of these physical effects may affect the stability of salidroside more obviously.

### 4.4. Microwave-Assisted Extraction (MAE) Method

The microwave-assisted extraction (MAE) method is to utilize microwave energy to rupture the cells, thus speeding up the dissolution of the active ingredients. MAE has the advantages of high extraction efficiency and short time. In 2021, Liu et al. [30] investigated the influence of the extraction time, extraction temperature, solid–liquid ratio, and solvent concentration on the extraction efficiency of salidroside. It was found that the optimal process parameters were 90 °C, 60% ethanol, and a solid–liquid ratio of 2 g/100 mL. The maximum extraction rate of salidroside was 2.17% within 20 min. The extraction efficiency of microwave-assisted extraction is sensitive to the extraction time, extraction temperature, solid–liquid ratio, and solvent concentration. For example, although a set of optimal parameters were obtained in Liu et al.’s study, in actual application, the raw materials of Rhodiola from different sources may need to be re-adjusted to achieve better extraction results due to factors such as composition differences, which increases the complexity of operation and the cost of preliminary testing.

### 4.5. Supercritical CO_2_ Extraction Method

Supercritical CO_2_ extraction has emerged as a cutting-edge green extraction method known for its high efficiency, environmentally friendly characteristics, and excellent selectivity. In recent years, this technique has been extensively utilized in combination with other methods for the extraction of salidroside. Wang et al. [31] investigated the impact of the supercritical CO_2_ extraction–microwave-assisted method and supercritical CO_2_ extraction–ultrasonic method on the extraction rate of salidroside. The results showed that the extraction rate of salidroside by the former method was 95.3%, while the latter method was 85.9%. It can be seen that the extraction of salidroside by the microwave method is significantly higher than that by the ultrasonic method under the same conditions. The supercritical CO_2_ extraction process depends on specific high-pressure conditions. Excessive pressure may lead to changes in intermolecular forces, deforming salidroside molecules or breaking chemical bonds, thus affecting their stability and activity. When supercritical CO_2_ extraction is combined with microwave or ultrasonic methods, although the extraction rate can be improved, the thermal effect and vibration effect brought by microwave and ultrasonic methods may further increase the influence on the stability of salidroside based on the original supercritical CO_2_ extraction.

### 4.6. Enzymatic Hydrolysis Method

Enzymatic hydrolysis decomposes plant cell walls into small molecules under the condition of an enzymatic hydrolysis reaction and accelerates the dissolution of the active ingredients of medicinal materials [32]. Wang et al. [33] optimized the process for the extraction of salidroside by cellulase-assisted ultrasonication using response surface methodology. The results showed that the optimal extraction conditions were an extraction time of 29 min, 0.18% cellulose, an extraction temperature of 43 °C, and a 39% ethanol concentration. The extraction rate of salidroside was 4.49 mg/g. Although the purpose of enzymatic hydrolysis is to break down the plant cell wall to facilitate the dissolution of the active ingredients, enzymes may cause some side reactions during the reaction. For example, enzymes may have unintended interactions with salidroside or other plant components, resulting in changes in the chemical structure of salidroside. This change may reduce the stability of salidroside, make it more susceptible to external factors (such as temperature, light, etc.), and may even lead to the loss of its activity.

### 4.7. Flash Extraction Method

The flash extraction method is a new type of extraction technology. It relies on high-speed mechanical shear force and hyperactive molecular percolation technology so that the active ingredients are rapidly extracted. Fan et al. [34] optimized the flash extraction process of salidroside based on a single-factor test, and the results showed that the optimal extraction parameters were a solid–liquid ratio of 1:20, extraction voltage of 140 V, extraction time of 40 s, and extraction times of two times (40 s/time). The extraction rate of salidroside was 3.83 ± 0.12%. Compared with the ordinary method, the results showed that flash extraction was 165.37% higher than ordinary extraction. The flash extraction method can achieve fast extraction, and multiple extraction operations can be performed in just 40 s of extraction time. Although this improves extraction efficiency, the rapid extraction process can cause salidroside to experience large environmental changes in a short period. For example, it is quickly transferred from the relatively stable plant cell environment to the extraction medium and is subjected to the combined action of many factors such as the mechanical shear force and electric field, which leads to the reduction in its activity.

### 4.8. Ultra-High-Pressure Extraction Method (HP-UHPEM)

Ultra-high-pressure extraction involves using the hydrostatic pressure of 100~1000 MPa on material liquid at room temperature. When the effective components reach the dissolution balance, they quickly relieve the pressure. Chen et al. [35] used the central composite design-response surface methodology to optimize the extraction process of salidroside under ultra-high pressure. The results showed that the optimal process parameters were an extraction solvent of 73.3% ethanol, pressure of 255.5 MPa, liquid–solid ratio of 29.5:1 (mL:g), and extraction time of 2 min. The extraction rate of salidroside was 9.29 mg/g, which was 31.6% higher than that of the reflux method, 20.3% higher than that of the ultrasonic method, and 9.7% higher than that of the microwave method. The ultra-high-pressure extraction method will quickly relieve pressure after the active ingredient reaches dissolution equilibrium. When the pressure is suddenly reduced, it may lead to structural instability of salidroside, such as the disordered arrangement of molecules and abnormal vibration of chemical bonds, which may affect the stability and activity of salidroside.

### 4.9. Multi-Technology-Coupling Extraction Method (MTCE)

In recent years, the multi-technology-coupling extraction method has been used frequently in the isolation and purification of natural products. Sun et al. [36] proposed a two-step ultra-high-performance liquid chromatography (UPLC) method consisting of DIAION HP-20 adsorption and silica gel column chromatography, which can simultaneously produce high-purity salidroside from the Rhodiola rhizome. The results showed that DIAION HP-20 could successfully remove impurities except for salidroside during gradient elution with 5~20% ethanol, and the purity of salidroside was 94.17% with an overall yield of 39.09%.

Above all, the solvent extraction method and enzyme hydrolysis method are suitable for large-scale production, but they are time-consuming. Ultra-high-pressure extraction, flash extraction, microwave extraction, and ultrasonic extraction have the advantages of a high rate of extraction, short time, and low energy consumption, but they have high requirements for extraction equipment. At present, there are few studies on the multi-technology-coupling extraction of salidroside. Therefore, it is necessary to continue to strengthen the research on more advanced and efficient multi-technology-coupling methods to greatly improve extraction efficiency.

## 5. The Synthetic Pathways of Salidroside

Due to the special growing environment of *Rhodiola*, large-scale excavation, and overgrazing, *Rhodiola* has become an endangered species [37]. As a result, the source of salidroside is also reduced. Therefore, the synthesis of salidroside, including chemical synthesis, plant biosynthesis, and engineering bacteria biosynthesis, has been studied to resolve the shortage of salidroside.

### 5.1. Chemical Synthesis

#### 5.1.1. The Glycosidation of 1-Bromo-Tetraacetyl Pyranose Was Promoted by Silver Salt

One of the earlier reports on the chemical synthesis of salidroside was published by Troshenko et al. in 1969 [38]. According to the structure of salidroside, the O-glucosidation of 2-(4-hydroxy-benzene) ethanol (1) and 1-bromo-tetraacetyl glucopyrase (2) was used to obtain the intermediate tetraacetyl salidroside by the Koenigs–Knorr method [39], and then saponification and deacetylation to prepare salidroside (A).

**Figure 5:**
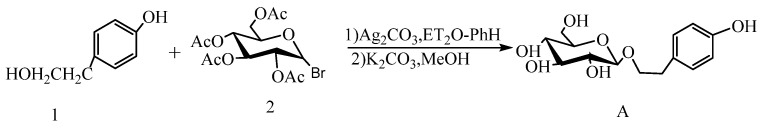
Synthesis of salidroside (A) by reaction of tyrosol (1) with bromotetraacetyl glucose (2).

In 1983, Ming et al. used the method in Figure 5 to synthesize salidroside [40], and the total productivity was 20%. In 1989, Endo et al. [41] also synthesized salidroside (A) by a similar method. In addition to the conditions in Figure 5, other alkaline conditions such as Na_2_CO_3_-MeOH, MeONa-MeOH, NH_3_-MeOH, or Et_3_N-MeOH can be used for the saponification deacetylation [42,43].

The above method belongs to the direct glycosidation of tyrosol, which can improve the purity of salidroside by protecting the phenol hydroxyl group in tyrosol from glycosidation.

**Figure 6:**
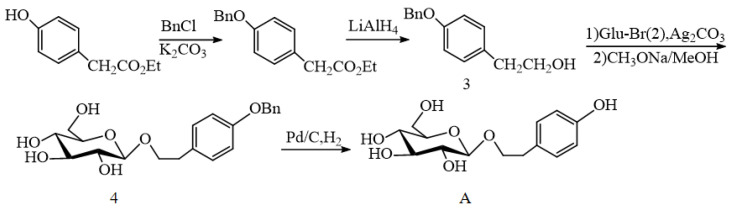
Dibenzyl protects salidroside (A) synthesis of the phenol hydroxyl group of tyrosol.

In 1996, Li et al. [44] used ethyl p-hydroxy phenylacetate as a raw material and simultaneously reduced it with lithium aluminum hydride to obtain benzyl-protected tyrosol. Then, applying the Koenigs–Knorr method, tyrosol was reacted with 1-bromotetraacetylglucose (Glu-Br, 2) under the promotion of Ag_2_CO_3_ to produce the intermediate tetraacetyl salidroside. The acetyl group was further removed with sodium methanol/methanol to produce salidroside with the phenolic hydroxyl group protected by benzyl group (4). Finally, the benzyl group was removed under Pd/C catalysis, and the total productivity of the salidroside was 70% (the last three steps) (Figure 6).

**Figure 7:**
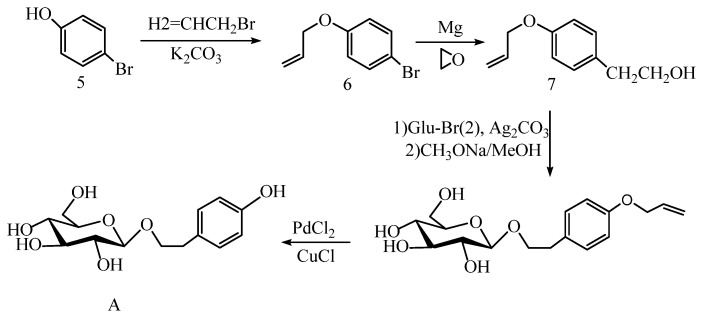
Synthetic strategy of salidroside (A) from p-bromophenol.

The tyrosol phenol hydroxyl group can also be protected by other groups. One year after the synthesis strategy of Li et al. [44], Zhang et al. [45] synthesized salidroside (A) in four steps using p-bromophenol as raw material (Figure 7). In the presence of K_2_CO_3_, p-bromophenol (5) is added to 3-bromopropylene to obtain compound (6), which is converted to 2-(4-allyl) ethanol (7) by the Grinnell reaction. Then, the productivity of salidroside with allyl-protected phenol hydroxyl was 65% using the Koenigs–Knorr method. Finally, allyl was removed by modified palladium chloride/copper chloride, and the total yield of salidroside (A) was 46%.

**Figure 8:**
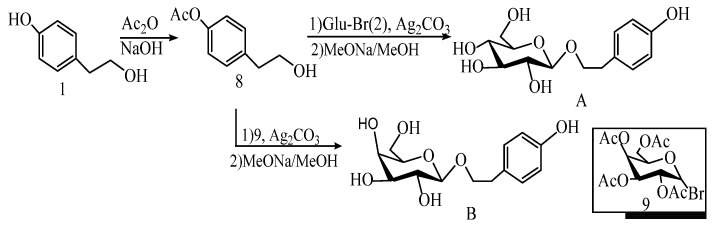
Synthetic strategy of salidroside (A) [46].

Shi et al. [46] synthesized salidroside by a two-step method in 2011. Firstly, the phenolic hydroxyl group of tyrosol was protected with acetyl groups to obtain 2-(4-methoxyphenyl) ethanol (8). Secondly, silver salts were used to promote the o-glycosylation reaction with 1-bromosugar and 9 (9 is a D-galactose derivative), respectively, using the acetyl group to protect the phenol hydroxyl group of tyrosol to obtain 2-(4-acetoxy-phenyl) ethanol (8), and then using silver salt to promote the O-glycosylation reaction with 1-bromose and 9 (9 is a d-galactose derivative), respectively (Figure 8). Salidroside and its derivative tyrosol galactoside can be obtained on a kilogram scale, and their total yield was above 72%. At the same time, the purity was over 98%.

#### 5.1.2. Salidroside Was Prepared by O-Glucosidation Reaction Promoted by Lewis Acid

**Figure 9:**
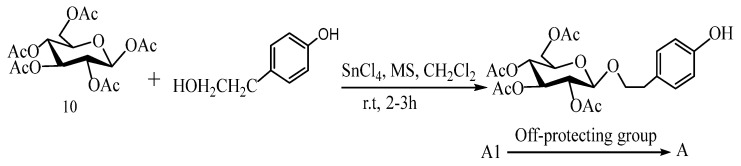
Synthetic strategies of salidroside (A) [47].

In 2007, Wu et al. [47] directly used readily available pentadactyl glucose (10) and aerosol as raw materials, catalyzed by Lewis acid SnCl_4_ and reacted at room temperature for 2 h to obtain salidroside in the presence of a 4Å molecular sieve, which was acetyl-protected by sugar, and then removed by alkaline saponification (MeONa-MeOH) to release salidroside (Figure 9). The total productivity was 44%. If the glycosylation reaction is catalyzed by Lewis acid BF_3_-Et_2_O, the total yield of salidroside is only 11%.

In 2011, Li et al. [48] reported a successful synthetic method for the glycosidation reaction using B-D-pentaethylphthaloylglucose as a donor and acyl-protected phenolic hydroxylated tyrosol as a glycosylated acceptor. The results showed that the O-glycoside productivity could reach 56% under the promotion of a single Lewis acid, and the influence of different Lewis acids on the productive rate was insignificant. However, it was observed that prolonging the reaction time tended to increase the productivity of O-glycosylation.

In 2016, some scholars [49] synthesized salidroside by using fall-acetyl glucose and 4-benzoxy phenyl ethanol as raw materials and Lewis acid and magnesium halide as catalysts, with an overall productivity of up to 63%. The process is simple, low-cost, and suitable for industrial large-scale production.

#### 5.1.3. Preparation of Salidroside by O-Glucosidation Catalyzed by Coordination Complex

**Figure 10:**
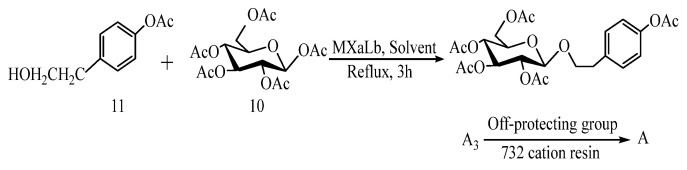
Synthesis strategy of salidroside (A) [50].

In 2017, Qiu et al. [50] studied the catalytic effect of transition metal coordination catalyst MXaLb on the O-glycosidation of 4-acetoxyphenylethanol (11) and pentaacetylglucose (10) (Figure 10). Under the action of the catalyst MXaLb, the metal M can be nickel (Ni) or palladium (Pd), X can be a halogen or acetyl, and L can be triphenylphosphine (Ph3P) or nitrile (CN). The results show that salidroside (A) can be obtained from all O-glycosidation reactions catalyzed by a coordination complex. However, considering factors such as the cost of total synthesis of salidroside (A), Ni should be selected as M in the catalyst MXaLb, Cl or OAc should be selected as X, and CN should be selected as L. In addition, high-boiling-point DMF and DMSO are not required as solvents. This method can avoid the trouble of preparing 1-position active group-substituted glucopyranose derivatives and control the total synthesis cost. At the same time, there is the prospect of developing large-scale total synthesis of salidroside (A).

Above all, chemical synthesis methods with low cost and fast speed can achieve the industrial production of salidroside, but there are problems: (1) In the glycosylation reaction of 1-bromo-tetraacetyl pyranose promoted by silver salt, silver salt is easy to decompose in the presence of air and light, so it is often needed for immediate use. In addition, the bromine reaction of the synthetic raw material 1-bromo-tetraacetyl glucopyranoside often leads to more pollution problems that need to be solved. (2) Salidroside is formed by the direct glucosidation of tyrosol. There are both phenolic and alcohol hydroxyl groups in tyrosol, and the phenolic hydroxyl group will be glycosylated together with the alcohol hydroxyl group, resulting in by-products that are difficult to remove. (3) It is very difficult to synthesize salidroside by reacting phenol hydroxyl-protected tyrosol with 1-bromo-tetraacetylglucopyranose. When using silver carbonate as a catalyst, excess silver carbonate is required to achieve a better catalytic effect, which is costly and unstable. To avoid residual silver impurities, column chromatography also needs to be used to remove impurities and purify the product. Therefore, the reaction conditions of chemical synthesis methods of salidroside are relatively strict, and the biosafety of this reaction is difficult to guarantee.

### 5.2. Plant Biosynthesis

**Figure 11:**
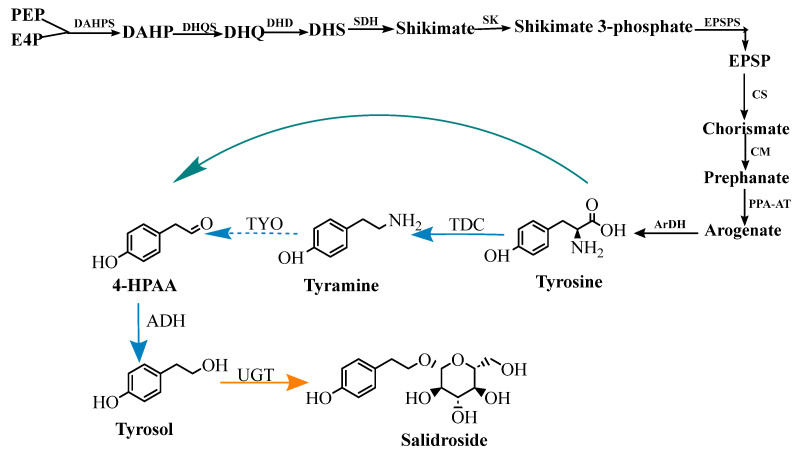
Biosynthetic pathway of salidroside.

The biosynthetic pathway of salidroside can be divided into three parts: the production of aromatic amino acids from phosphoenolpyruvate (PEP) and erythrose-4-phosphate (E4P) by shikimic acid and aryl acid pathways (Figure 11, black line); conversion of aromatic amino acids into tyrosol (Figure 11, green line and blue line); and subsequent glycosylation reaction (Figure 11, yellow line) [51].

**Figure 12:**
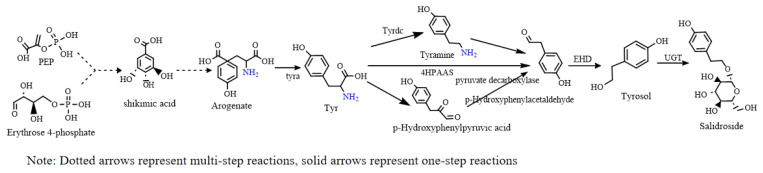
Proposed biosynthetic pathways of salidroside [56].

In recent years, the biosynthetic pathway of tyrosol and the development of the natural uridine diphosphate-glucosyltransferase (UGT) enzyme (uridine diphosphate glycosyltransferase) have been the focus of attention [52]. Li et al. [53] measured the content of salidroside from Rhodiola kirilowii and L-phenylalanine ammonia-lyase (PAL), cinnamate-4-hydroxylase (C4H), and tyrosine ammonia-lyase (TAL) activity, and found significant differences between salidroside content and the activity of the three metabolic enzymes. The researchers speculated that the synthesis of salidroside was sometimes dominated by tyrosine metabolism and sometimes by phenylalanine metabolism under different temperature culture conditions. At present, most researchers believe that the precursor of salidroside and tyrosol is tyrosine, and it is speculated that tyrosine can produce tyrosol in three ways, as shown in Figure 12. Tyrosine was catalyzed by tyrosine decarboxylase to form tyramine, and monoamine oxidase catalyzed tyramine to produce 4-hydroxyphenylac-etaldehyde (4-HPAA), which in turn was reduced to form tyrosol by alcohol dehydrogenase. Tyrosine is catalyzed by tyrosine aminotransferase to produce 4-hydroxyphenylpyruvate (4-HPP), which decarboxylates p-hydroxyphenylpyruvate to p-hydroxyphenylacetal, and then p-hydroxyphenylacetal is reduced to tyrosol [54]. Tyrosine can directly, through the 4-hydroxyphenyl acetaldehyde synthase (4-HPAAS), catalytically generate hydroxyl benzene acetaldehyde, then after the reduction reaction, generate tyrosol [55].

**Figure 13:**
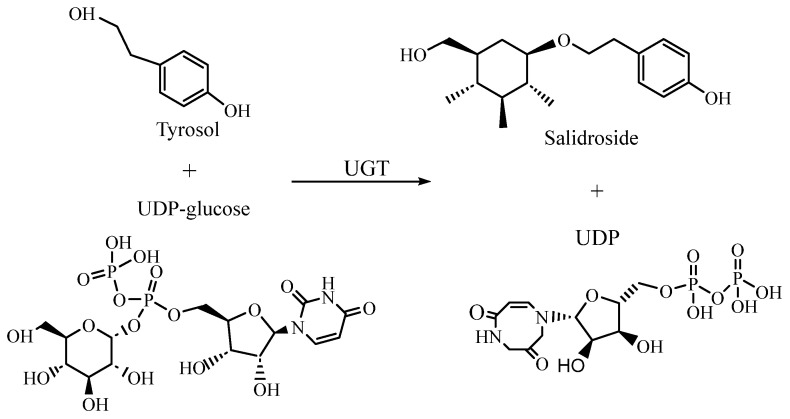
The last reaction of salidroside biosynthesis.

UGTs act as the key enzymes for converting tyrosol to salidroside in the downstream pathway. In 2007, a putative UGT cDNA from *R. Sachalinensis* A. Bor, termed UGT73B6 [57], was isolated, which is the first reported UGT gene associated with salidroside synthesis. Under the UGT, tyrosol reacts with uridine diphosphate-glucose (UDP-glucose) to produce salidroside and uridine diphosphate (UDP). An in vitro enzyme activity experiment and transgenic hairy root system of *Rhodiola* were used. It has been proven that UGT74R1 and UGT72B14 from *R. Sachalinensis* A. Bor also have the ability to convert tyrosol to salidroside [58]. After the overexpression of UGT72B14, UGT74R1, and UGT73brward6 in hairy roots of transgenic sea ginseng, the erythrocyte content increased to 420%, 50%, and 130%, respectively, indicating that UGT72B14 had the highest activity against tyrosin. To reveal the complete biosynthetic pathway of salidroside, 34 UGT genes were analyzed. The results showed that four UGTs (RrUGT 17, 29, 32, and 33) had region-specific T8GT activity, which could convert tyrosol into salidroside, of which RrUGT33 had the highest activity (Figure 13) [59].

### 5.3. Engineering Bacteria Biosynthesis

With the development of genetic engineering technology, the clarification of the salidroside biosynthesis pathway, and the clarification of rate-limiting enzymes in a metabolic pathway, the research on the synthesis of salidroside by microbial engineering bacteria has made breakthrough progress; especially the research using model microorganisms (such as *Escherichia coli* and c) as hosts has developed rapidly.

*Escherichia coli* has a short reproductive cycle, simple operation, and clear genetic background; so, in recent years, more researchers have selected *Escherichia coli* as a chassis organism for appropriate modification to produce salidroside. In 2011, Yu et al. reported that three UGTs of *R. Sachalinensis* A. Bor were recombinantly expressed in *Escherichia coli*, and the products of *Rhodiola* were obtained by an enzymatic reaction of recombinant enzymes in vitro. In 2014, Bai et al. [60] introduced key enzymes such as *Saccharomyces cerevisiae* ARO10 and UGT73B6 of *R. Sachalinensis* A. Bor, adopted the strategy of cell growth and product synthesis to fermentate recombinant *Escherichia coli* in different media segments, and introduced UGT73B6, a glycosyltransferase from *R. Sachalinensis* A. Bor. The yield of salidroside with glucose as a substrate was 56.9 mg/L. This is the first report of de novo synthesis of salidroside in *Escherichia coli*. To further improve the selectivity and catalytic efficiency of UGT and the conversion rate of tyrosol, in 2017, Chung et al. [61] introduced parsley AAS and Arabidopsis UGT85A1, and also cultured recombinant *Escherichia coli* with glucose as a substrate by segmentalization to increase the yield of salidroside to 288 mg/L. In the same year, Fan et al. [62] further obtained the highly active Bacillus licheniformis UGTBL1 through gene mining and constructed the recombinant *E. coli*. After whole-cell catalysis of the engineered bacterium for 24 h, the yield of salidroside could be increased to 1.04 g/L. In 2019, Chen et al. [63] obtained a salidroside high-yielding strain by modifying endogenous proteins and expressing ARO10p from *Saccharomyces cerevisiae* and AtUGT85A1 from *Arabidopsis thaliana*, with salidroside yields up to 9.30 g/L.

All of the above are the strategies for producing salidroside with a single engineered strain. With the development of microbial co-culture technology, researchers began to try to use microbial co-culture technology to produce salidroside. In 2018, Liu et al. [64] constructed two strains of *Escherichia coli* by introducing key genes such as *Saccharomyces cerevisiae* ARO10, pichia pastoris KDC4, and arabidopsis UGT85A1, and adopted the co-culture mode and batch-feed regulation strategy of the two strains. The yield of salidroside was further increased to 6.03 g/L by fermentation with glucose and xylose as substrates for 129 h.

With the technology of synthesizing salidroside from *Escherichia coli* maturing, people began to focus on the research and development of synthesizing salidroside from *Saccharomyces cerevisiae*. In 2018, Torrens-Spence et al. [65] established the synthetic pathway of salidroside in *Saccharomyces cerevisiae* by introducing salidroside 4HPAAS and T8GT and adopted the codon optimization strategy to obtain the yield of salidroside at 1.5 mg/L. In the same year, Jiang et al. [66] used gene integration technology to integrate aro4K229L, aro7G141S, aroL, pcaas, and atugt85a1 into yeast chromosomes in two steps to establish a genetically stable strain. This was the first plasmid-free salidroside synthetic strain constructed, and the yield of salidroside was 732.5 mg/L after fermentation for 168 h by the method of fed-batch fermentation. In 2021, Liu et al. [67] conducted a comprehensive transformation of *Saccharomyces cerevisiae*, and the modified strain obtained 26.55 g/L salidroside, which is the highest yield reported so far.

Salidroside arouses particular and persistent interest because of its significant biological activities as a water-soluble phenolic natural product. The synthesis of salidroside is mainly through chemical synthesis and biosynthesis. The overall comparison shows that the yield of some improved chemical synthesis methods is higher, which can reach more than 70%, but there are problems such as complex reaction conditions, high cost, and pollution. The yield data of plant biosynthesis are lacking, and it is limited by many complex factors such as the plant growth cycle and enzyme activity. The biosynthesis yield of engineered bacteria is improved rapidly and has great potential, from the initial tens of mg/L to the current 26.55 g/L, showing a good development prospect in large-scale industrial production. Among them, the synthetic methods using *Escherichia coli* and *Saccharomyces cerevisiae* as hosts have made remarkable progress. In the future, the yield and quality of salidroside can be increased, the production cost can be reduced, and large-scale industrial production can be achieved by optimizing the strains and improving the fermentation process.

## 6. Derivatives of Salidroside

Salidroside has multiple hydroxyl groups and strong polarity, which makes it difficult to penetrate the plasma membrane and the blood–brain barrier, resulting in low membrane permeability and digestion and absorption [68]. Many studies have found that the acylation of the natural glycosides of salidroside can improve its bioavailability and pharmacological properties. In addition, by changing the structure of phenolic glycosides to balance their lipophilicity and hydrophilicity, the membrane permeability of salidroside can be enhanced and its bioavailability can be improved.

**Figure 14:**
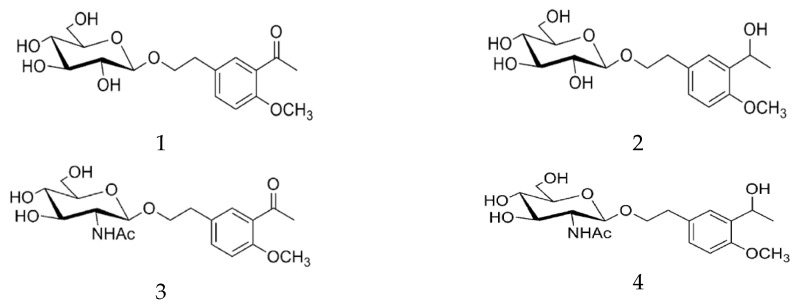
Structure of salidroside derivatives. Note: Compounds 1-4 are all salidroside derivatives.

In recent years, the potential neuroprotective effect of salidroside has attracted more and more attention. It has been reported that salidroside can protect PC12 cells from glutamate-induced excitatory toxicity or SH-SY5Y human neuroblastoma cells from H_2_O_2_-induced apoptosis [69,70]. To improve the permeability and neuroprotective effect of salidroside, some scholars have found that salidroside derivatives containing the acetyl group can enhance the neuroprotective effect on the damage caused by glucose and serum depletion, and inhibit the induced apoptosis. By coupling the phenyl ring of salidroside with acetyl or its reducing group, four target compounds were designed and synthesized (Figure 14: 1–4), and it was confirmed that four compounds had protective effects on cell viability, especially compound 1, which had the most significant protective effect compared with salidroside [71].

**Figure 15:**
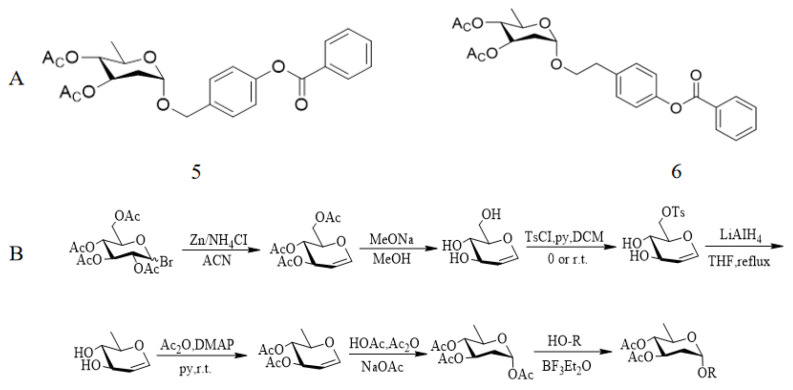
Synthetic route of salidroside derivatization [73]. Note: (**A**) The dianalogues of salidroside were screened as compounds 5 and 6 according to (**B**); (**B**) The synthetic route of salidroside analogues.

In addition, salidroside can protect cardiomyocytes due to its antioxidant and anti-inflammatory effects and is considered as one of the potential compounds for the treatment of myocardial damage in sepsis [72]. However, the anti-inflammatory activity of salidroside is low and its pharmacokinetic properties are not ideal. To improve its anti-inflammatory activity and promote clinical application, a series of salidroside derivatives were synthesized as shown by the Figure 15B method, and the structure of phenolic glycosides was changed. The effects of compounds 5 and 6 were screened, and the phenolic hydroxyl groups in compounds 5 and 6 were esterified to further improve their bioavailability (Figure 15A). When both are absorbed into the blood, the phenol hydroxyl group is released due to the catalytic action of esterase, increasing its anti-inflammatory activity. To further evaluate the anti-inflammatory activity of the compound, the levels of IL-1β, IL-6, and TNF-α of RAW264.7 cells stimulated by lipopolysaccharides were detected by an ELISA kit for 24 h. The results showed that compound 5 and compound 6 down-regulated the expression of IL-1β, IL-6, and TNF-α in a dose-dependent manner. Finally, its anti-inflammatory activity was also confirmed in a rat model of septic myocardial injury. It is concluded that compounds 5 and 6 have good therapeutic effects on septic myocardial injury, and they may be candidates for anti-inflammatory and anti-septic myocardial injury [73].

Many studies have shown that salidroside derivatives also have good anti-inflammatory, anti-hypoxia, and central nervous system protection activities. So, it is very important to conduct the chemical synthesis, structural derivation, and structural optimization of salidroside. Some derivatives or analogs of salidroside may be structurally modified to enhance their binding affinity with the target and improve their bioavailability, to achieve efficient treatment. For example, the chemical modification of the glycosylated part of salidroside or the benzene ring structure may change its pharmacokinetic characteristics, making it easier to absorb, distribute, and metabolize in the body, and reach the target of action faster and play a role.

## 7. Pharmacological Effects of Salidroside

*Rhodiola* is a kind of medicinal and edible plant, which is widely used to prevent altitude sickness. Salidroside is one of the main active ingredients of *Rhodiola*. A growing number of studies have found that salidroside has various pharmacological activities and has great potential in the treatment of central nervous system diseases, cardiovascular diseases, tumors, and other diseases. Therefore, this section systematically summarizes the pharmacological effects and related mechanisms of salidroside and provides a reliable basis for the subsequent clinical research and safety of salidroside.

### 7.1. Activity on the Central Nervous System

**Figure 16:**
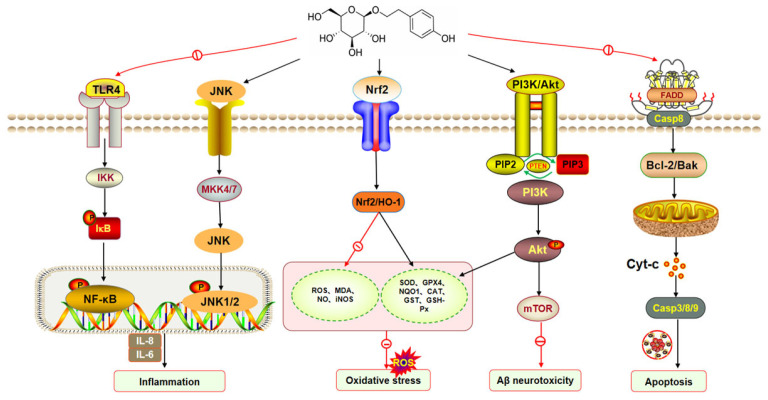
The central nervous system protects the activity of the salidroside.

Central nervous system (CNS) diseases, which include neurodegenerative diseases, traumatic brain injuries, and mental disorders, are characterized by serious damage to the brain or spinal cord. The clinical features of CNS diseases are diverse, and their treatments are intricate [74]. Recent studies have demonstrated that salidroside has neuroprotective effects on CNS diseases [75,76]. The biological effects and their corresponding mechanisms are clearly illustrated in Figure 16.

#### 7.1.1. Anti-Alzheimer’s Disease Effect

Alzheimer’s disease (AD), a primary form of dementia, is characterized by memory loss and a gradual decline in cognitive function [77]. Salidroside has a certain inhibitory effect on inflammation and oxidative stress in the development of AD [78].

Oxidative stress plays a crucial role in the development of AD. ROS-induced oxidative stress attacks nerve cells in specific regions of the brain, leading to decreased levels of SOD and GPX4, increased levels of MDA, and accelerated apoptosis in hippocampal neurons [79]. Salidroside is a natural antioxidant, which can improve oxidative stress-induced neuronal network dysfunction by scavenging ROS and inhibiting cell apoptosis. In an experiment of amyloid-beta (Aβ)-induced AD PC12 cells, salidroside (12.5, 25, 50, 100, 200 μmol/L) enhanced the viability of cells in a dose-dependent manner, decreased the levels of ROS and MDA, increased the levels of SOD, and up-regulated the phosphorylation levels of ERK1/2 and Akt. It is suggested that salidroside alleviates neuronal oxidative stress by activating ERK1/2 and Akt signaling pathways [80]. Mitochondria, as the primary site of ROS production, are particularly vulnerable to oxidative stress-induced damage. After 75 days of treatment with 50 mg/kg salidroside for AD mice models, it was found that salidroside could significantly improve mitochondrial membrane density and potential, and up-regulate the expression of GPX4 and HO-1, thus inhibiting the production of ROS and alleviating oxidative stress damage. These potential mechanisms are associated with salidroside activating the Nrf2/HO-1 pathway [81].

The inflammatory response is the driving force to induce the occurrence and development of AD and is also the main target of AD treatment. Studies have shown that the expression of toll-like receptor 4 (TLR4) is significantly elevated in the AD brain. The activation of TLR4 can mediate NF-κB, further promote the expression and release of inflammatory factors, and aggravate the inflammatory damage of neurons. Gao et al. found in the AD rat model induced by the subcutaneous injection of D-galactose (120 mg/kg) that salidroside (20, 40 mg/kg) could significantly reduce the levels of TNF-α, IL-1β, and IL-6 in the hippocampus, and down-regulate the expression of p-Iκbα, p-IKKα, and p-IKKβ. The mechanism is related to the salidroside regulating the NF-κB signaling pathway and reducing hippocampal inflammatory response. The above results were also confirmed in a rat model of AD established by the intraperitoneal injection of scopolamine, suggesting that salidroside can repair the pathological damage of hippocampal neurons by regulating the central inflammatory response [82,83].

#### 7.1.2. Anti-Parkinson’s Disease Effect

Parkinson’s disease (PD) is one of the common neurodegenerative diseases in a clinic. Patients have movement disorders, including bradykinesia, resting tremor, muscle rigidity, and neuropsychiatric disorders [84]. The abnormal accumulation of α-synuclein is one of the important causes of dopaminergic loss and dysfunction in PD, which has strong neurotoxicity. The activation of the Nrf2/ARE signaling pathway can inhibit the excessive accumulation of α-synuclein [85]. In the process of inducing PD mice models by the intraperitoneal injection of 1-methyl-4-phenyl-1-2-3-6-tetrahydropyridine (MPTP), salidroside (30, 60 mg/kg) could up-regulate TH, Nrf2, and NQO1 expression and down-regulate α-synuclein expression in the brain. After knocking down Nrf2, the protective effect of salidroside was weakened or even disappeared, suggesting that salidroside can inhibit the excessive accumulation of α-synuclein by mediating the Nrf2/ARE signaling pathway for the treatment of PD [86]. NLRP3 inflammation promotes the secretion of IL-1β and IL-18 and the formation of GSDMD pores by activating caspase 1, which leads to the apoptosis of PD brain neurons and aggravates the process of neurodegeneration [87]. Zhang et al. [88] used salidroside to interfere with MPTP-induced PC-12 and LPS-induced BV2 in vitro and found that 2, 10, and 50 μmol/L salidroside could effectively increase TH and decrease α-syn expression. At the same time, the expressions of IL-1β, IL-18, and GSDMD were down-regulated by inhibiting the TXNIP/NLRP3/Caspase-1 signaling pathway. It was proven that salidroside can reduce the damage of dopamine neurons by regulating the NLRP3-related apoptosis pathway, and play a neuroprotective role in PD.

Mitochondrial autophagy is an important pathway to remove abnormal proteins and repair them. PINK1/parkin-mediated mitophagy plays a key role in alleviating MPTP-induced nerve injury [89]. Loss of substantia nigra neurons leads to dopamine deficiency in the substantia striatum nigra (SNpc), which is one of the neuropathological features of PD. Using MPTP-C57BL/6 mice to simulate the PD model, it was found that the expression of autophagy-related proteins LC-3β and Beclin1 was increased and the expression of PINK1 and parkin was decreased in SNpc. After treatment with salidroside (30, 50 mg/kg), the above damage was significantly improved, suggesting that salidroside enhances mitochondrial autophagy through the activation of the PINK1/parkin pathway to ameliorate the neurological damage of PD [90]. Excessive oxidative stress can lead to the death and apoptosis of dopaminergic neurons, which in turn promotes the onset and development of PD [91]. In experiments using the MPTP/MPP-induced PD PC12 cell model, it was found that 10, 20, 40, and 80 μmol/L salidroside could dose-dependently increase the level of GSH, and decrease the level of ROS and MDA and the level of apoptosis-related proteins caspase-9, caspase-3, and Bax in PC12 cells. The results suggest that salidroside may exert neuroprotective effects on PD through antioxidant stress response [92]. Rho A promotes the activation of ROCK II, which leads to the transfer of microglia to the site of inflammation and mediates the release of various inflammatory factors, leading to dopaminergic neuronal damage and accelerating the progression of PD [93]. Zhou et al. [94] established a PD mouse model using paraquat. After treatment with 10 and 20 mg/kg salidroside, DOPAC and DA levels in the striatum of the mice increased, while TNF-α, IL-6, IL-1β, NK-κB, ROCK II, and Rho A in the brain tissue significantly decreased. The results indicated that salidroside could inhibit the activation of NK-κB by regulating the Rho/ROCK II pathway to reduce the neuroinflammatory response.

#### 7.1.3. Anti-Cerebral Ischemia

Ischemic stroke (IS) is a pathology in which multiple factors cause the narrowing or blockage of intracerebral arteries, followed by pathological damage to the brain such as ischemia, hypoxia, neuronal necrosis, and neurological dysfunction [95]. Cerebral ischemia–reperfusion injury (CIRI) [96] is one of the major secondary injuries of IS, which can lead to permanent disability or even death in severe cases. Apoptosis is one of the major forms of neuronal loss during cerebral ischemic injury, and excessive apoptosis induces the formation of homodimers of Bcl-2-associated X-protein (Bax) and Bcl-2 antagonist factor (Bak), which translocate to the mitochondria and activate caspase-3, thereby exacerbating neurological dysfunction [97]. Zhang et al. [98] found that the apoptosis rate of nerve cells in the brain tissue of rats with total CIR was significantly elevated, the expression of p53 and Bax was increased, and the expression of Bcl-2 was decreased. Salidroside (12, 48 mg/kg) significantly reversed the expression of the above proteins, suggesting that salidroside may reduce the apoptosis of nerve cells by inhibiting p53 and its downstream apoptotic factors. Using the HT22 cell model induced by oxygen–glucose deprivation followed by reperfusion (OGD-R) as a research object, it was found that salidroside could increase Bcl-2/Bax levels and PARP protein expression and inhibit the expression of pro-caspase-9 and pro-caspase-3. It is suggested that salidroside can inhibit the activation of caspase-3 and reduce PARP cleavage to block neuronal apoptosis [99]. In addition, glycogen synthase kinase-3β (GSK-3β), as a multifunctional serine/threonine kinase, can regulate the opening of mitochondrial mPTP and promote caspase-9-induced neuronal apoptosis during CIR. In the middle-cerebral-artery occlusion/reperfusion (MCAO/R) rat model, it was found that the intraperitoneal injection of salidroside (50 mg/kg) could reduce the apoptosis of nerve cells by reducing the protein expression of phosphorylated (p)-GSK-3β and caspase-9 in brain tissue [100].

Oxidative stress is the main factor of secondary nerve injury after cerebral ischemia. A large amount of ROS in neurons accumulates during CIRI, leading to lipid peroxidation, mitochondrial membrane destruction, and other pathological damage. Salidroside (intravenously injected, 5~24 mg/kg) can significantly increase the contents of SOD, GSH-PX, and GSH; decrease the contents of induced nitric oxide synthase (NOS), NO, and MDA; and protect neurons from oxidative stress injury in focal cerebral ischemia–reperfusion rats [101]. Nuclear transcription factor E2-related factor (Nrf2) activated by extracellular-regulated protein kinase 1/2 (ERK1/2) also plays an important role in cerebral ischemic injury. When oxidative stress occurs, Nrf2 in the cytoplasm dissociates with Kelch-like ECH-associated protein (Keap), phosphorylates, and migrates to the nucleus, where it binds to the antioxidant response elemental sequence (ARE) and promotes the expression of the antioxidant gene [102,103]. Han et al. [104] injected salidroside intraperitoneally into CIRI rat models before and after perfusion, respectively. The results showed that salidroside could not only increase the activity of SOD, GST, and GSH-Px in the cerebral cortex and striatum, and reduce the content of MDA, but also up-regulate the expression of Nrf2 and its downstream enzyme HO-1. This suggests that the salidroside may play an antioxidative stress role through the ERK1/2/Nrf2/HO-1 signaling pathway and its downstream antioxidant enzymes.

Cerebral ischemia injury is closely related to inflammation, and different inflammatory cascades play different roles in cerebrovascular injury states. CD44 and CD14 are monogenic-encoded glycoproteins on the surface of cell membranes, which are mainly expressed in neutrophils and can promote the inflammatory response and lymphocyte activation after cerebral ischemic injury [105]. Using the wire embolization method to replicate the middle cerebral artery occlusion (MCAO) model in rats, salidroside (50 mg/kg) was injected intraperitoneally and continuously for 6 d. The results showed that salidroside could reduce the expression of microglial markers, CD44 and CD14, in the cerebral infarction surrounding area; increase the ratio of p-Akt to total Akt; and up-regulate the expression of HIF-α, thus reducing inflammatory infiltration in ischemic brain tissue [106,107]. Liu et al. [108] established a microglia cell line (BV2) OGD/R model and a CIRI rat model and found that salidroside (6.25~100 μM; 50 mg/kg) can reduce the expression of NLRP3, NF-κB, IL-1β, and IL-18 in ischemic brain tissue. It is suggested that the inhibition of the TLR4/NF-κB/NLRP3 signaling pathway is one of the mechanisms.

#### 7.1.4. Anti-Depressive Effects

The mechanism of depression is related to the imbalance of brain neurotransmitters such as norepinephrine and 5-hydroxytryptamine (5-HT) [109]. Qin et al. [110] treated a chronic stress-induced depression rat model with salidroside (ig. 1.5 g/kg) for 3 weeks. Similarly to the positive control group (fluoxetine), salidroside significantly decreased the level of 5-HT in the hippocampus and increased the number of 5-bromodeoxyuracil (Brd U)-labeled cells and the expression of β-tubulin III. It is suggested that salidroside can alleviate depression by inhibiting 5-HT levels and promoting the proliferation and differentiation of hippocampal neural stem cells. Recent evidence suggests that neuroinflammation is a key mediator of depression, and, in particular, the expression of pro-inflammatory factors such as NLRP3 and IL-1β is significantly enhanced in depressed patients [111]. A mice model of depression was induced by the subcutaneous injection of 20 mg/kg corticosterone (CORT) and the continuous intragastric administration of 40 mg/kg salidroside for 21 days [112]. It was found that the level of BDNF protein in the hippocampus was significantly up-regulated, and the levels of NF-κB, NLRP3, and IL-1β were significantly decreased, suggesting that salidroside may ameliorate depression by inhibiting the NF-κB/NLRP3 signaling pathway. In addition, the toll-like receptor 4 (TLR4) signaling pathway is involved in the regulation of the body’s immune response and is closely related to the immune inflammatory process of depression. Mo et al. [113] found in an LPS (1 mg/kg)-induced inflammatory depression mice model that salidroside (ig 25 mg/kg, administered for 2 w) could significantly improve depression-like behavior (sugar water preference test, forced swimming test, suspension test), and down-regulate TNF-α, IL-1β, TLR4, and NLRP3 levels in brain tissue. It is indicated that salidroside regulates inflammation by mediating the TLR4 signaling pathway, which is one of the mechanisms of action in the treatment of depression. Other studies have shown that the cAMP/PKA/CREB signaling pathway is widely involved in a series of neuropathological reactions of depression, including abnormal regional brain activity, altered synaptic function, and impaired neurogenesis [114]. During the construction of a depressed rat model using chronic unpredictable mild stress (CUMS), salidroside (ip 12.5, 25, 50 mg/kg, administered for 6 w) significantly attenuated hippocampal nerve damage, and inhibited the rate of neuronal apoptosis and the expression of IL-1β, IL-6, and TNF-α levels in the hippocampus of CUMS rats. Meanwhile, cAMP, PKAc-II, and p-CREB/CREB protein expressions were up-regulated. It is suggested that salidroside may inhibit the inflammatory response and neuronal apoptosis through the cAMP/PKA/CREB signaling pathway [115].

#### 7.1.5. Cognition Enhancement

Cognitive dysfunction encompasses a range of cognitive impairments caused by different factors. Postoperative cognitive dysfunction (POCD) is a common complication after major surgery, with the neuroinflammatory response playing a pivotal role in its development [116]. Tang et al. [117] conducted a study using a rat model of POCD induced by the internal fixation of a tibia fracture. They found that administering salidroside (at a dose of 75 mg/kg for seven days) reduced the levels of IL-6, TNF-α, MCP-1, and the cannabinoid type 2 receptor (CB2R) in serum. This suggests that salidroside may improve postoperative POCD by potentially reducing hippocampal CB2R expression and mitigating inflammatory responses. The central nervous system is susceptible to hypoxia, which can lead to varying degrees of damage under prolonged hypoxic conditions, resulting in memory loss and cognitive dysfunction [118]. Yang et al. [119] utilized a low-pressure oxygen chamber to simulate the plateau environment and create a cognitive failure model of hypoxic plateau rats. Following 28 days of chronic hypoxia, they discovered that administering salidroside (at a dosage of 1 g/kg intraperitoneally) significantly increased SOD and GSH-Px activities while decreasing MDA content in the hippocampus. This indicates that salidroside may enhance the cognitive performance of plateau hypoxic rats by inhibiting oxidative stress. Vascular dementia (VD) is characterized by cognitive dysfunction resulting from the blockage of large blood vessels and the diffusion of small vessel disease [120]. To induce the VD rat model through bilateral common carotid artery ligation, salidroside was administered for 29 days (at 20 mg/kg intraperitoneally). Salidroside notably decreased ATP levels and mitochondrial membrane potential, ultimately enhancing mitochondrial function. This suggests that it is one of the mechanisms of action of salidroside to improve mitochondrial activity [121]. Moreover, diabetes-induced cognitive impairment is a complication of diabetes characterized by cognitive disorders and structural changes in the brain [122]. Using a rat model of diabetic cognitive dysfunction induced by the intraperitoneal injection of streptozotocin (at 60 mg/kg), Hao et al. [123] observed that administering salidroside (at 200 mg/kg intragastrical for four weeks) significantly improved cognitive dysfunction, while reducing the expression levels of IL-6 and TNF-α. Further investigations revealed that salidroside prevents diabetes-induced cognitive dysfunction by modulating the Rho/ROCK/SIRT1/NF-κB pathway.

#### 7.1.6. Anti-Epileptic Effect

Epilepsy is a type of chronic recurrent disease caused by sudden abnormal brain discharge, leading to cognitive impairment and activity disorders. The onset of epilepsy may be worsened by oxidative stress and chronic inflammation [124]. In a rat model induced with pentylenetetrazol to mimic epilepsy, the administration of salidroside [intraperitoneal, 50 mg/kg for 30 days] reduced the secretion of IL-1β and TNF-α by activating the Nrf2/antioxidant response element (ARE) pathway. Salidroside exhibits anticonvulsant and neuroprotective effects by protecting vertebral neurons in the CA3 region, thereby inhibiting the abnormal firing process seen in epilepsy [125]. In another rat model of epilepsy induced by pilocarpine and lithium chloride, intraperitoneal injections of salidroside (0.5, 1.0, and 1.5 g/kg for 30 days) resulted in increased levels of catalase (CAT) and SOD and decreased GSH, while reducing the expression of cleaved caspase-12, cleaved caspase-3, C/EBP homologous protein (CHOP), and glucose-regulated protein 78 (GRP78) in hippocampal tissue [126]. These results suggest that salidroside can inhibit the apoptosis of hippocampus cells in an epileptic state, and the mechanism of action may be related to inhibiting oxidative stress and endoplasmic reticulum stress.

#### 7.1.7. Spinal Cord Injury

Spinal cord injury (SCI) is a serious neurological disease. Inflammation can lead to secondary damage, aggravating tissue degradation, and loss of function in SCI [127]. Li et al. [128] conducted a study using a rat model of SCI and intraperitoneally administered salidroside (10, 20, and 40 mg/kg) for 28 days. The findings showed that salidroside can notably reduce mRNA levels of NF-κB, iNOS, and COX-2; elevate p-AMPK; and decrease p-mTOR expression. These results indicate that salidroside can reduce neuroinflammation and restore motor function by regulating the AMPK/mTOR signaling pathway. Additionally, Su et al. [129] observed that salidroside (200 μg/mL) inhibited NF-κB, ERK, and p38 protein activation in LPS-induced astrocytes, suggesting that salidroside may modulate inflammatory responses following spinal cord injury by targeting the NF-κB, p38, and ERK signaling pathways. Previous research has emphasized the role of oxidative stress in SCI progression. Liu et al. [130] found increased levels of lipid peroxide MDA and decreased SOD activity in the tissue of rats with SCI. After the intraperitoneal injection of salidroside (20 mg/kg) for seven days, these indicators had been markedly reversed. A further analysis revealed that salidroside may alleviate oxidative stress and promote healing by activating the Nrf2/HO-1 signaling pathway in the spinal cord.

#### 7.1.8. Anti-Huntington’s Disease Effect

Huntington’s disease (HD) is a hereditary neurodegenerative disorder characterized by a progressive decline in neuronal function, with its pathological manifestations closely associated with the aggregation of polyglutamine (PolyQ) [131]. In a study by Xiao et al. [132], they utilized PolyQ-induced Cryptomeria japonica as an HD model. They observed that treatment with salidroside at concentrations of 50~200 μM for three days reduced ROS levels, increased antioxidant enzyme activity, and decreased neuronal death. These findings suggest that salidroside may offer neuroprotection against the detrimental effects of PolyQ by mitigating oxidative stress.

Above all, salidroside plays a role in the prevention and treatment of CNS diseases through various mechanisms such as anti-inflammation, antioxidative stress, the inhibition of apoptosis, and the protection of mitochondrial function. Among them, there are many studies on anti-inflammatory and antioxidant mechanisms, which may be a breakthrough point for the treatment of CNS diseases in the future.

### 7.2. Activity on the Cardiovascular System

**Figure 17:**
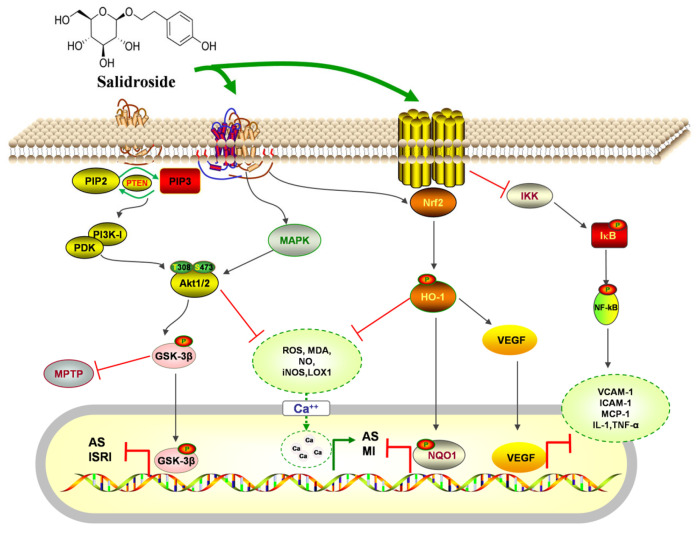
The cardiovascular system protection activity of salidroside.

Cardiovascular disease, a significant health issue with a complex pathogenesis, is widely recognized as one of the burdensome diseases in society. It encompasses ischemic or hemorrhagic conditions affecting the heart and even the entire body, often stemming from factors like hyperlipidemia, blood viscosity, atherosclerosis, and hypertension. Recent research has highlighted the potential of salidroside in protecting the cardiovascular system and preventing cardiovascular diseases through various pathways, including anti-atherosclerosis function, anti-myocardial cell injury function, improvement of vascular function, and hemorheology. The biological effects and underlying mechanisms are clearly illustrated in Figure 17.

#### 7.2.1. Anti-Atherosclerosis

Atherosclerosis (AS) is recognized as the primary pathological basis of cardiovascular diseases. Pathological research has confirmed that endothelial damage, inflammatory response, oxidative stress, and the activation of vascular smooth muscle cells are related to the development of AS [133]. Among these factors, vascular inflammation plays a crucial role in influencing the progression of AS. VCAM-1, ICAM-1, and MCP-1 can jointly affect the migration of white blood cells across the vascular endothelial cells (ECs) and the accumulation at sites of inflammation. It further damages the vascular ECs and increases their permeability, promoting the formation of AS plaques [134]. Using mice fed a high-fat diet for 12 weeks to replicate the AS model, the results demonstrated that salidroside (50 mg/kg) significantly reduced serum lipid levels and plaque area from the aortic arch to the abdominal aorta. The mechanism of action may be related to down-regulating the levels of VCAM-1, ICAM-1, and MCP-1, thus inhibiting the inflammatory reaction during the formation of AS [135]. Oxidative stress can lead to endothelial dysfunction and further aggravate AS. Human umbilical vein endothelial cells (HUVECs) were used to construct the AS model and incubated with salidroside (0.1, 1, and 10 μM) for 24 h. The results showed that salidroside reduced ROS and MDA levels and increased SOD and CAT activities. The mechanism may be related to activating the Nrf2/HO-1 signaling pathway, up-regulating the expression of NQO1, and protecting ECs from oxidative stress damage [136].

Foam cell formation and apoptosis play a crucial role in the pathogenesis of AS. Studies have shown that ATP-binding cassette transporter A1 (ABCA1) facilitates the release of free cholesterol from monocyte macrophages to apolipoprotein A1, promoting reverse cholesterol transport [137]. At the same time, lectin-like oxidized lipoprotein receptor-1 (LOX1) can lead to lipid plaque instability and exacerbate AS formation. Ni et al. [138] in THP1 human acute monocytic leukemia cells induced by oxidized low-density lipoprotein (ox-LDL) demonstrated that salidroside (0.1, 1, and 10 μM) dose-dependently increased the expression of ABCA1 and decreased the expression of LOX1, effectively inhibiting foam cell formation and enhancing plaque stability. In addition, it was found that salidroside can induce the nuclear translocation of Nrf2 by activating the MAPK/AKT signaling pathway, thus preventing the occurrence of apoptosis of foam cells.

#### 7.2.2. Anti-Myocardial Cell Injury

Myocardial cell injury involves reversible and irreversible damage, with apoptosis and necrosis being the main types of myocardial injury. Recent studies have shown that salidroside has protective effects on myocardial cells. H_2_O_2_-induced H9c2 cells were used as a research object and incubated with salidroside (10^−6^, 10^−5^, 10^−4^ mol/L) for 24 h. The results showed that salidroside can significantly reduce the release of LDH, CK, and MDA, while increasing the activity of the antioxidant enzyme SOD, indicating that salidroside could improve myocardial cell injury by inhibiting oxidative stress [139]. In another study, it was also found that salidroside can effectively inhibit the production of intracellular MDA and protect cardiomyocytes from oxidative damage. The specific mechanism may be related to activating the PI3K/Akt pathway and enhancing antioxidant enzyme activity [140].

Mitochondria play a crucial role as a site of intracellular redox reactions. Impairment of mitochondrial function in cardiomyocytes can readily disrupt cellular energy metabolism, resulting in myocardial injury [141]. Yin et al. [142] used H_2_O_2_-induced H9c2 cells to replicate the myocardial injury model and treated them with 25, 50, 75, and 100 μmol/L salidroside for 10 min. The results show that salidroside can protect against H_2_O_2_-induced myocardial damage through the mitochondrial pathway, and this protective effect may be achieved by activating the PI3K/Akt signaling pathway to promote the phosphorylation of GSK-3β, thereby inhibiting the opening of mPTP.

In addition, salidroside has the effect of antagonizing intracellular calcium overload, thus protecting cardiomyocytes. Zhang [143] established an H_2_O_2_-induced injury model on primary cultured SD rat cardiomyocytes and found that salidroside (10, 30, and 90 μg/mL, incubation for 2 h) could reduce MDA content, inhibit LDH activity, and increase SOD activity, while reducing the concentration of Ca^2+^ and the rate of cardiomyocyte apoptosis. This suggests that salidroside has an inhibitory effect on apoptosis, and the mechanism of action may be related to the inhibition of intracellular calcium overload and lipid peroxidation.

#### 7.2.3. Improvement of Vascular Function

Salidroside can increase coronary blood flow and cardiac output, and reduce coronary and systemic resistance, suggesting that salidroside can affect vasoconstriction and diastolic function. Rabbit thoracic aortic rings were used for a study, and 1 μmol/L norepinephrine (NE) was administered to stimulate vasoconstriction to a maximal level while salidroside (10^−11^~10^−6^ mol/L) was added at intervals of 5 min. The results showed that salidroside could dilate the vasoconstriction induced by NE in a dose-dependent manner. It was further found in primary cultured bovine thoracic aorta endothelial cells that salidroside (10^−4^, 10^−5^, 10^−6^ mol/L) could increase the tNOS activity in the lysate of endothelial cells and promote the release of NO after incubation for 8 h, indicating that salidroside can exert a vasodilator effect by regulating the NO pathway [144]. In recent years, studies have shown that salidroside can alleviate the damage of cytokines to blood vessels. A total of 50 mg/kg salidroside could inhibit the overexpression of NF-κB, IL-1, TNF-α, E-selectin, and intercellular adhesion molecule-1 and reduce pulmonary vascular exudation by way of tail vein injection, suggesting that salidroside could inhibit hypoxia-induced pulmonary vascular inflammation and vascular permeability.

Vascular Endothelial Growth Factor (VEGF), fibroblast growth factor (FGF), and endothelin are involved in regulating the angiogenesis process in the body. Angiogenesis can effectively relieve myocardial ischemia and protect the cardiovascular system. The rat model of acute myocardial infarction was established by coronary artery ligation, and 10, 20, and 40 mg/kg salidroside were given for 4 weeks. The results showed that salidroside can significantly up-regulate the expression of VEGF in the ischemic myocardium, indicating that salidroside promotes angiogenesis in the myocardium by up-regulating VEGF expression [145,146].

#### 7.2.4. Improvement of Hemorheology

Hemorheological abnormalities are an increase in whole blood viscosity and a slowing down of blood flow in microcirculation, which participate in the formation of cardiovascular diseases [147]. In recent years, salidroside has been found to have the effects of anti-platelet aggregation, lowering the viscosity of whole blood, and regulating blood lipids. Wei et al. [148] found that salidroside (5.0, 10.0 mg/kg) can significantly reduce the erythrocyte specific volume, blood viscosity, and platelet aggregation rate, and prolong coagulation time, indicating that salidroside has an anti-thrombosis effect. The mechanism of action is related to improving the rheological properties of blood.

Above all, salidroside plays an important role in the prevention and treatment of cardiovascular diseases. However, there are unclear relationships between the signaling pathways and targets involved in the cardiovascular protection process of salidroside. In addition, the study on the intervention of salidroside in hemorheology is relatively rare and the research in this field should be further strengthened.

### 7.3. Anti-Inflammatory

Inflammation is an important defense mechanism for the body against harmful stimuli such as tissue damage and infection; however, an excessive inflammatory response can lead to body dysfunction. It has been confirmed that the anti-inflammatory activity of salidroside can be used in the treatment of various diseases and the mechanism of action is related to the inhibition of the NF-κB signaling pathway [149]. Gao et al. [150] used anterior cruciate ligament transection (ACLT)-induced osteoarthritis in rats as a research subject and found that salidroside (12.5, 25, 50 mg/kg) inhibited the phosphorylation of NF-κB and decreased TNF-α, IL-17, and VCAM expression, thus alleviating the inflammatory response and promoting chondrocyte proliferation. Using cecum ligation and puncture to replicate the sepsis mice model, it was found that salidroside (80, 160, and 320 mg/kg intraperitoneally) could down-regulate the expression of NF-κB p65 and p38 MAPK, suggesting that salidroside has a protective effect on intestinal barrier dysfunction by inhibiting the NF-κB/p38 MAPK signaling pathway [151]. In the brain inflammation of mice induced by LPS, salidroside (50 mg/kg) can reduce neutrophil infiltration and inhibit the expression of NF-κB, TNF-α, and IL-1β by activating the PI3K/Akt pathway, thus playing an anti-inflammatory role [152].

The expression and release of inflammatory factors mediated by the JAK/STAT pathway is a key trigger of hepatocyte injury. In plateau hypoxia-induced liver injury experiments in rats, it was found that salidroside (20 mg/kg) significantly down-regulated the expression of IL-1β, TNF-α, IL-6, and MCP-1, while reducing the levels of p-JAK2 and p-STAT3, suggesting that the inhibition of the JAK2/STAT3 pathway by salidroside is one of the anti-inflammatory mechanisms of its action [153]. In addition, the interaction of Nrf2 with Keap1 can directly inhibit the activation of Nrf2, which in turn reduces the production of inflammatory mediators. A rat model of bronchopneumonia was established by the intranasal injection of lipopolysaccharides (1 mL/g), and it was found that salidroside (30, 60 mg/kg) significantly reduced the number of eosinophils, neutrophils, and lymphocytes in alveolar lavage fluid, and down-regulated the expression of IL-1β, iNOS, and Keap1, while up-regulating the expression of Nrf2 in the lung tissues, suggesting that salidroside may alleviate the inflammatory response of bronchopneumonia by activating the Nrf2/Keap1 signaling pathway [154].

**Figure 18:**
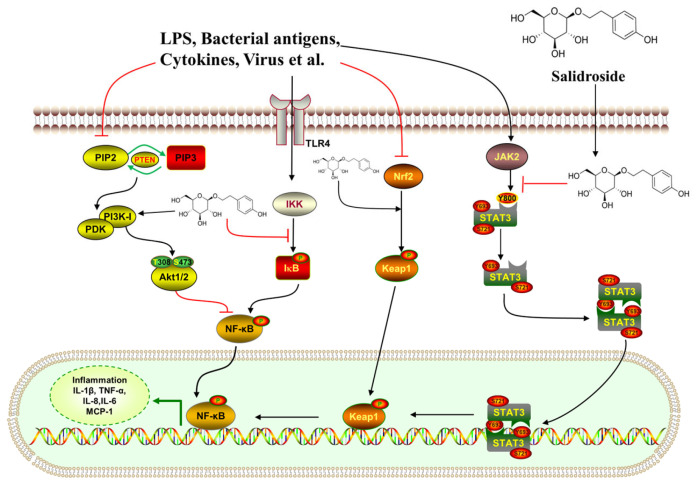
The anti-inflammatory activity of salidroside.

Above all, salidroside exerts anti-inflammatory effects through multi-target and multi-pathway activities and has broad application prospects. However, most studies have focused only on the NF-κB signaling pathway, ignoring the linkage effect with other signaling networks. Therefore, follow-up studies should further investigate the anti-inflammatory mechanism of salidroside. The biological effects and their underlying mechanisms are visually represented in Figure 18.

### 7.4. Anti-Fibrosis

Fibrosis is the result of multiple types of tissue damage, especially in the course of chronic inflammatory-responsive diseases. In recent years, it has been found that salidroside has a significant therapeutic effect mainly on pulmonary, hepatic, and renal fibrosis.

#### 7.4.1. Anti-Pulmonary Fibrosis

Pulmonary fibrosis is a chronic interstitial lung disease characterized by the progressive proliferation of fibrous tissue. A rat lung fibrosis injury model was induced by a single intratracheal instillation of bleomycin (BLM) at 5 mg/kg, and the results showed that salidroside (50, 100, and 200 mg/kg, intraperitoneal injection for 28 days) dose-dependently inhibited the IκBα phosphorylation and the NF-κB p65 nuclear translocation and activated the Nrf2 signaling pathway. It is speculated that salidroside against pulmonary fibrosis may be related to the activation of Nrf2 and the inhibition of the NF-κB signaling pathway [155]. MMPs/TIMPs are the main enzyme system regulating ECM degradation, and an imbalance between MMP-2 and TIMP-1 expression is a key link in the onset and progression of pulmonary fibrosis. The BLM-induced pulmonary fibrosis model in rats was further treated with salidroside for 14 d. The results showed that the salidroside could up-regulate TIMP-1 and reduce the expression of MMP-2, and the degree of lung tissue lesions, suggesting that the salidroside could inhibit the process of pulmonary fibrosis by regulating the balance of MMP-2/TIMP-1 [156].

#### 7.4.2. Anti-Hepatic Fibrosis

Hepatic fibrosis refers to the pathological response caused by the diffuse over-deposition and abnormal distribution of ECM during the liver injury–repair process, the central point of which is the activation of hepatic stellate cells (HSCs) [157]. Carbon tetrachloride (CCl_4_) was used to induce hepatic fibrosis in a mice model, while 10 and 20 mg/kg salidroside were injected intraperitoneally for 8 weeks. It was found that salidroside could inhibit the production of ECM and the activation of HSCs by blocking the TGF-β1/Smad3 signaling pathway to alleviate liver fibrosis [158]. The hepatic stellate cell line JS1 was cultured in vitro and given 10^−5^ mol/L salidroside for 24 h. The results showed that salidroside inhibited CXCL16-induced cell migration and a high expression of Col I and α-SMA mRNA, and the phosphorylation of Akt, indicating that salidroside can attenuate hepatic fibrosis, and the mechanism may be related to the inhibition of CXCL16-induced migration and Akt phosphorylation [159].

#### 7.4.3. Anti-Renal Fibrosis

Renal fibrosis is the main pathological change in chronic kidney disease progressing to end-stage renal failure. Epithelial–mesenchymal transition (EMT) is the primary mechanism leading to renal fibrosis [160]. During the replication of a rat model of renal fibrosis using the intraperitoneal injection of streptozotocin, salidroside (50, 100, and 200 mg/kg) significantly inhibited the expression of collagen IV, α-SMA, and N-cadherin, and induced the expression of E-cadherin in rat kidney tissues. It was further found that its mechanism of action may be associated with the inhibition of the TGF-β1/Smad pathway [161]. In addition, Shati et al. [162] found that salidroside could significantly reduce the total levels of Wnt1, Wnt3, and β-catenin in rats with renal fibrosis, suggesting that salidroside may play an anti-renal fibrosis role by inhibiting the Wnt1/Wnt3a/β-catenin signaling pathway.

In general, salidroside has significant therapeutic advantages for pulmonary fibrosis, hepatic fibrosis, and renal fibrosis, and is a drug with potential for clinical application. However, there are fewer studies overall, especially for myocardial fibrosis. Therefore, it is still necessary to systematically evaluate the mechanism of anti-fibrotic efficacy of salidroside in the future, to provide new ideas for the clinical treatment of fibrotic diseases.

### 7.5. Anti-Tumor

Malignant tumors cause great harm to human health. Studies have shown that salidroside has obvious anti-tumor effects, including the inhibition of tumor cell proliferation, induction of tumor cell apoptosis, blocking of tumor cell metastasis, and enhancement of body immune function.

#### 7.5.1. Inhibition of Tumor Cell Proliferation

The inhibition of tumor cell proliferation is one of the key aspects of tumor treatment. Zhang et al. [163] incubated gastric cancer cells (NU-GC-3) with 10 and 20 μg/mL salidroside for 24 h and found that salidroside up-regulated the expression of TGF-β1, and blocked the binding of cyclin E and cyclin to the CDK2, CDK4, and CDK6, suggesting that salidroside may inhibit the proliferation of NU-GC-3 cells by promoting the expression of TGF-β1. In addition, Wang et al. [164] investigated the effects of salidroside (1, 5, 10, and 20 µg/mL) on A549 lung cancer cells and found that salidroside significantly inhibited TGF-β-induced tumor invasion and markedly reduced tumor cell proliferation. The mechanism may be through the inhibition of the ROS/p38MAPK signaling pathway.

#### 7.5.2. Induction of Tumor Cell Apoptosis

Apoptosis is the programmed death of cells, in which the pro-apoptotic gene Bax and the anti-apoptotic protein Bcl-2 play important roles. In the study of lung adenocarcinoma A549 cells, salidroside (50 μg/mL) can significantly induce apoptosis, promote the expression of apoptosis proteins and genes such as Bax, and inhibit the expression of anti-apoptosis proteins and genes such as Bcl-2. The mechanism of action may be related to the down-regulation of Wnt/β-catenin signaling pathway. In an experimental study on human colorectal cancer cells (HT-29 cells), salidroside (0.5, 1, and 2 mM incubation for 48 h) dose-dependently decreased the expression rate of Bcl-2/Bax and inhibited the phosphorylation of PI3K, Akt, and mTOR, suggesting that salidroside induced apoptosis of human colorectal cancer cells by inhibiting the PI3K/Akt/mTOR pathway [165,166,167].

#### 7.5.3. Blocking Tumor Cell Metastasis

The disruption of the dynamic balance of angiogenic factors in tumor tissue stimulates the emergence of neovascularization. This process prompts tumor cells to infiltrate and metastasize in the surrounding area [168]. Liu et al. [169] investigated the effects of salidroside (12.5, 25, 50, 100, and 200 μmol/L incubation for 24 h) on the migration of C6 glioma cells. It was found that salidroside blocked the migration of tumor cells by down-regulating the expression of MMP-9, reducing neovascularization, and increasing cell adhesion. Salidroside (0.5, 1.0, and 2.0 mg/mL) was co-cultured with human hepatocellular carcinoma HepG2 cells for 24 h, 48 h, and 72 h, and the results showed that salidroside could dose-dependently reduce the adherence of HepG2 cells to the basement membrane, and down-regulate the expression of MMP-1, suggesting that salidroside could inhibit the infiltration and metastasis of tumor cells by regulating MMP-1 expression [170].

#### 7.5.4. Enhancement of the Immune System

Tumor initiation and growth are correlated with the body’s immune function. Consequently, enhancing the body’s immune function may exert a specific inhibitory effect on tumor cells. Wang et al. [171] showed that salidroside (50, 100, and 200 mg/kg) could significantly increase the IgG and IgM contents in cervical cancer mice and enhance the non-specific immune function. In addition, salidroside (500 mg/kg) can also improve the local specific cellular immune function in mice with lung cancer and indirectly enhance the killing ability of splenic T-lymphocytes, which improves the cellular immune function [172].

To sum up, salidroside exerts anti-tumor effects through various pathways. However, most of them are in vitro experiments, and in vivo, studies of salidroside against tumors were carried out at a later stage.

### 7.6. Anti-Hypoxia

Hypoxia is a pathological process in which abnormal changes occur in the metabolism, function, and even morphological structure of tissues and cells when the body does not receive sufficient oxygen [173]. Recent studies have shown that mTOR, as a cellular nutrient-sensing and energy-regulating factor, is closely associated with hypoxia-induced injury [174]. Using a cobalt chloride (CoCl_2_)-induced hypoxic injury model in PC12 cells, the result found that salidroside (30, 60, and 90 μmol/L) increased the viability of PC12 cells, and promoted the phosphorylation level of mTOR and the downstream protein 4EBP1. This suggests that salidroside may attenuate hypoxia-induced injury by activating the mTOR/4EBP1 pathway [175]. Endothelin-1 (ET-1) is a vasoconstrictor produced by vascular endothelial cells. Teng et al. [176] co-incubated 40, 80, and 120 μg/mL salidroside with chicken embryonic pulmonary artery smooth muscle cells (PASMCs) for 1 h, followed by hypoxic storming for 24 h. The results revealed that salidroside significantly inhibited the expression of ET-1, ETA, and ETB, indicating that the anti-hypoxic effect of salidroside may be related to the down-regulation of the expression of ET-1 and its receptor. The levels of VEGF and HIF-1α were significantly increased in the organism under a hypoxic environment, suggesting that the expression of VEGF and HIF-1α was regulated by hypoxia [177]. During the establishment of the hypoxia rat model using a low-pressure and low-oxygen chamber, 32 mg/kg salidroside was injected intraperitoneally for 4 weeks. The results showed that salidroside reduced the pulmonary artery pressure (mPAP), right ventricular systolic pressure (RVSP), and right ventricular hypertrophy index in hypoxic rats, and the inhibition of the VEGF/HIF-1α signaling pathway was one of the mechanisms of anti-hypoxia effects [178]. In addition, hypoxia activates the immune response by regulating the expression and function of toll-like receptors (TLRs), which in turn causes the release of numerous inflammatory mediators. In CoCl_2_-induced hypoxic human renal tubular epithelial cells (HKCs), salidroside (25, 50, and 100 μg/mL) was found to down-regulate the expression of TLR2, and thus alleviate hypoxia-induced injury [179].

Mitochondria supply oxygen, ATP, and ion exchange for cellular energy metabolism. Tian et al. [180] used cardiomyocytes from mice after hypoxia/reoxygenation and incubated them with 5 μmol/L salidroside for 2 h. The results showed that salidroside could protect cardiomyocytes from hypoxia/reoxygenation by elevating the mitochondrial membrane potential and decreasing the release of mitochondrial cytochrome C.

**Figure 19:**
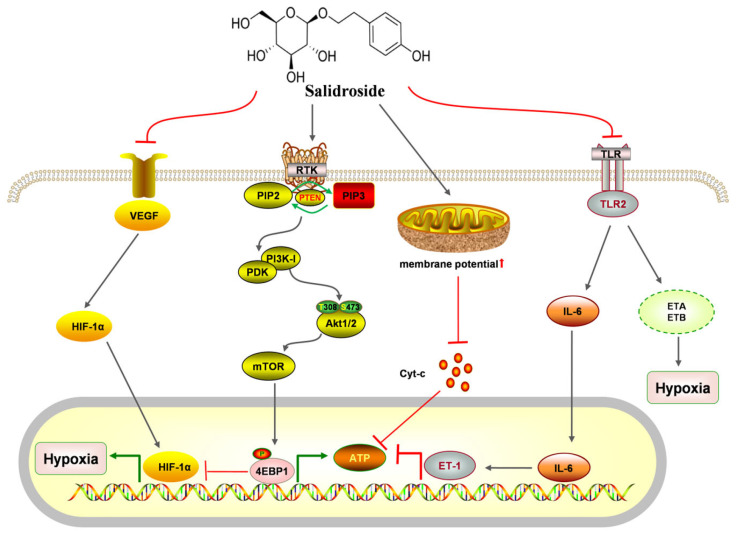
The hypoxia activity of salidroside.

Above all, salidroside exerts anti-hypoxic effects by modulating VEGF/HIF-1α and mTOR/4EBP1 pathways. In recent years, various kinds of plateau diseases caused by plateau hypoxia have constrained the development of the economy and society in the plateau area. Salidroside is expected to become a key drug for anti-plateau hypoxia. However, there are fewer studies on anti-plateau hypoxia injury, which should be strengthened subsequently. The biological effects and underlying mechanisms are clearly illustrated in Figure 19.

### 7.7. Anti-Aging

Aging is a degenerative change that occurs gradually in the body. Salidroside can inhibit the expression of aging markers and the level of oxidative stress to slow down the aging process [181]. During aging-like remodeling of rat cerebral vessels, salidroside (100 mg/kg) was found to significantly reduce the expression of aging biomarkers P65 and P16. Krüppel-like factor 4 (KLF4) overexpression can induce the down-regulation of anti-aging gene expression. Using homocysteine (Hcy)-induced aging in human umbilical vein endothelial cells (HUVECs), Zhang et al. found that salidroside increased cell viability and telomerase activity and inhibited the expression of p53, p21, and KLF4 mRNA in a dose-dependent manner, suggesting that salidroside’s inhibition of Hcy-induced cellular aging may be related to the down-regulation of KLF4 expression [182]. In addition, salidroside (0.2 and 1.0 g/kg) was found to increase the activities of SOD and GSH-Px and decrease the levels of LPO and lipofuscin in a natural-aging mice model, suggesting that salidroside delays aging by increasing the activities of endogenous antioxidant enzymes [183].

**Figure 20:**
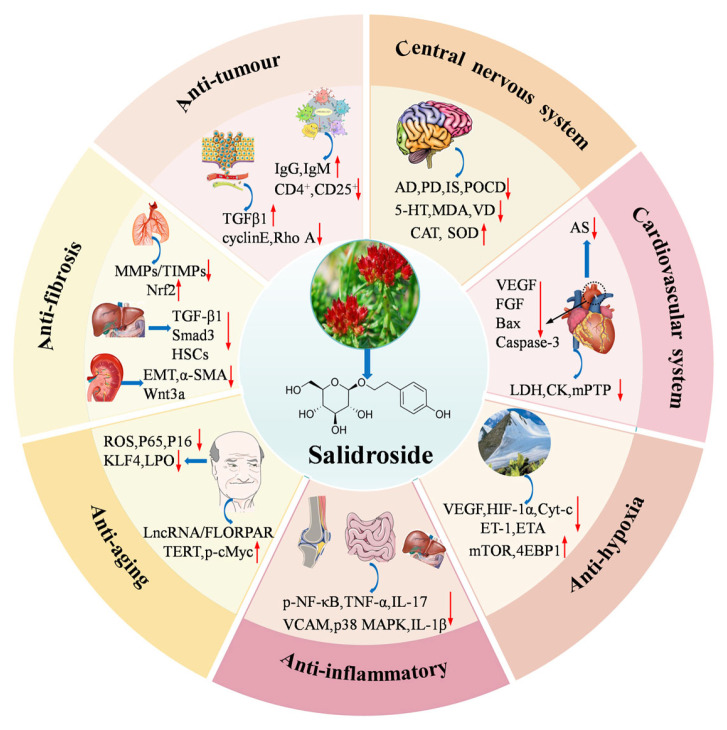
Pharmacological profiles of salidroside. Note: "↑" indicates an increase and "↓" indicates a decrease.

Aging is one of the hotspots of current research [184]. Salidroside has anti-aging activity, but their mechanism of action is not clear. In addition, the establishment and evaluation indexes of aging models are not perfect. The pharmacological profiles of salidroside are visually represented in Figure 20.

## 8. Pharmacokinetics of Salidroside

It is commonly known that herbal medicines have biological effects that are greatly influenced by how they are absorbed, distributed, metabolized, and excreted after administration.

The absorption: Gu et al. [185] studied a rat model of myocardial ischemia and found that after a single intra-abdominal injection of 50 mg/kg salidroside, the AUC_0–8h_ and C_max_ of the model rats were 0.35 and 0.39 times lower than that of normal rats, respectively. Salidroside has unique pharmacokinetic properties in rats with myocardial ischemia. After continuous administration for 7 days, the mean AUC_0–8h_ and C_max_ of salidroside were 2.89 times and 2.61 times higher than that of model rats after a single administration, and even 2.28 times and 4.03 times higher than that of the normal control group. In addition, studies have shown that the rat gut becomes saturated during salidroside absorption, and the sodium-dependent transport mechanism of glucose may be involved in this process [186].

The distribution: Zhang et al. [187] found that salidroside exhibited linear pharmacokinetic properties at intravenous doses of 7.5 mg/kg, 15 mg/kg, and 30 mg/kg. Plasma levels of salidroside were always higher than tissue levels after administration. In addition, concentrations of salidroside were higher in skeletal muscle than in the liver, brain, and fat. The distribution of salidroside in the ovary and testis was higher than that in the kidney and spleen.

The metabolism: Guo et al. [188] found that after intravenous administration, salidroside can be converted to p-tyrosol and may be further metabolized to sulfates glycosidic couplings, or methyl compounds. This process can lead to undetectable levels of tyrosol in plasma samples.

The excretion: Bao et al. [189] investigated the pharmacokinetics and tissue distribution characteristics of salidroside in mice. The results showed that the concentration of salidroside was higher in the plasma and liver tissue of mice 30 min after administration. After 1 h, the concentration of salidroside in plasma gradually decreased, and the distribution in liver and kidney tissues gradually increased, indicating that salidroside may be mainly excreted by the kidney.

## 9. Clinical Application and Safety

### 9.1. Clinical Application

Rhodiola grows in a special environment and therefore has a high pharmacological and health value. Nowadays, the active ingredients of *Rhodiola* (mainly salidroside) have been utilized in medicines, food, and healthcare products [190]. Using the database of the China Food and Drug Administration (CFDA), a total of eight *Rhodiola*-related proprietary Chinese medicinal products were found (Table 2), including Dazhu *Rhodiola* injection (two specifications), Dazhu *Rhodiola* tablets, narrow-leaf *Rhodiola* tablets, Dazhu *Rhodiola* capsules, compound Mountain *Rhodiola* Oral liquid, *Rhodiola* oral liquid, Mountain *Rhodiola* Oral liquid, and compound *Rhodiola rosea* oral liquid. Furthermore, there are 12 sizes of health products of *Rhodiola* (Table 3), and they all have the efficacy of promoting blood circulation and removing blood stasis, clearing the pulse and relieving pain. Meanwhile, it was found that the above-mentioned medicines have significant preventive and curative effects on cardiovascular diseases, neurological diseases, and respiratory diseases. Che et al. [191] confirmed that *Rhodiola rosea* injection reduces postoperative ischemia–reperfusion injury in patients with acute myocardial infarction by improving vascular endothelial function, inhibiting oxidative stress and inflammatory response. An alpine *Rhodiola rosea* oral solution (taking 10 mL twice a day for 8 weeks) can shorten the duration of angina pectoris, and the angina interval, and accelerate the blood flow velocity of the main coronary artery blood-supplying arteries, the peak systolic velocity, and the peak diastolic velocity, to effectively alleviate the clinical symptoms of angina pectoris in coronary artery disease [192]. In the process of treating patients with radiological lung injury, after 8 weeks of taking a compound alpine *Rhodiola rosea* oral solution (60 mg/time, three times/day), the levels of TGF-β1 and TNF-α were significantly reduced, and the levels of lung capacity (VC), forceful lung capacity (FVC), forceful expiratory volume in 1 s (FEV1), and FEV1/FVC were significantly increased, which suggests that the use of a compound alpine Rhodiola rosea oral solution in treating radiological lung injury may be related to the level of serum inflammation factors [193]. In addition, when *Rhodiola rosea* capsules were used to treat patients with Alzheimer’s disease, it was found that Rhodiola rosea capsules (taking two capsules three times a day for 63 days) could significantly increase the ADL score and the levels of SOD and GSH-Px, suggesting that the supplemental treatment of Alzheimer’s disease with *Rhodiola rosea* capsules has better efficacy and higher safety [194].

Although *Rhodiola* and its active ingredients have been developed into a series of products, there are still constraints in the development and utilization, such as the insufficient basic research on pharmacodynamic substances, little exploration of food and drug value, single product form, insufficient extension of the industrial chain, and lack of professional processing technology. In a later study, we can systematically study the basis of pharmacological substances in different parts, evaluate their safety and effectiveness, and clarify their food and medicinal value.

### 9.2. Safety

In recent years, the occurrence of herbal medicine poisoning has been widely discussed. Salidroside is the main active ingredient in *Rhodiola*. However, there are few studies on the toxicity of salidroside, and it is generally believed that salidroside is well tolerated in animals. For example, Zhu et al. [195] showed no maternal toxicity, embryotoxicity, or teratogenicity of salidroside injection in SD rats at doses of 0.5, 0.25, and 0.125 g/kg. Similarly, Cao et al. [196] also observed long-term toxicity of salidroside in rats. In this study, 80 rats were divided into four groups, of which three groups were administrated an intragastric administration at 0.2, 1.0, and 5.0 g/kg, respectively, to detect peripheral blood imaging and liver and kidney function. The results showed that there was no significant difference between the administration group and the control group. In addition, a 2012 study also showed that the acute application of *Rhodiola* extract in mice did not result in death, and there was no significant weight loss or changes in organs and microscopic tissues such as the heart, liver, and kidneys over 14 days. These findings suggest that Rhodiola extract is relatively safe. Cytochrome P-450 is an important metabolizing enzyme that has an impact on the toxicological significance of drug use [197]. Philip G et al. [198] found that when salidroside concentrations exceeded predicted plasma concentrations, <2 μM, there was no risk of drug interaction due to the Cyp-450, MAO enzyme, or OATP drug transporter. The combination of salidroside with other drugs should be given high priority due to potential drug interactions in terms of pharmacokinetics and pharmacodynamics. In conclusion, the above studies strongly support the low-toxicity potential of salidroside.

However, it is important to note that despite the low toxicity of salidroside, there may be some limitations to its therapeutic potential. One possible limitation is its low bioavailability. Low bioavailability means that only a small proportion of the administered salidroside may reach the target site in the body, reducing its efficacy. This could limit its use in certain therapeutic applications where high drug concentrations at the target site are required. Additionally, the lack of comprehensive long-term studies on human subjects is another concern. While animal studies provide some indication of safety, human physiology and metabolism can differ, and long-term effects in humans may not be accurately predicted by animal data.

The combination of salidroside with other drugs should be given high priority due to potential drug interactions in terms of pharmacokinetics and pharmacodynamics. In conclusion, the above studies strongly support the low-toxicity potential of salidroside, but its therapeutic application should be considered with an awareness of possible limitations.

## 10. Conclusions and Outlook

In summary, salidroside is a bioactive ingredient with high medicinal value. This review systematically summarizes the isolation and purification methods, different synthetic strategies, pharmacological effects and molecular mechanisms, pharmacokinetics, clinical applications, and safety of salidroside, which opens a new avenue for the large-scale production of salidroside and its derivatives. Meanwhile, it also strongly confirms the great potential of salidroside in medicinal chemistry and biomedicine. Although significant progress has been made in the research of salidroside in recent years, there are still some shortcomings:

(1) To date, drugs containing salidroside alone have not been used in clinical practice. The reasons for this may be related to the scarcity of wild Rhodiola resources and the low extraction rate and limited purity of the existing extraction methods, which make it difficult to meet the market demand. (2) Although the safety and tolerability of synthetic salidroside are considerable, the expensive catalysts and activation reactions of chemical groups during chemical synthesis need to be considered. (3) Salidroside has a wide range of pharmacological activities, but the specific targets of its effects are not clear, and most of the work is still in the basic research stage. (4) When salidroside is administered in the traditional way, the effective content of salidroside in the body is low, and the elimination in the body is fast. (5) Due to the lack of toxicological studies, the experimental data are not convincing enough, so long-term toxicity studies are needed to provide strong safety assurance for the clinical application of salidroside. (6) In terms of drug interactions, although some studies have shown that there is no risk of drug interaction related to certain drug transporters when the concentration of salidroside exceeds a certain range, the overall research on drug interactions is still not comprehensive enough, and there may be undiscovered risks of adverse reactions when using drugs in combination.

Given the above deficiencies, the synthesis technology of salidroside should be further optimized, and the mode of administration and pharmacological properties should be explored. In addition, rational clinical application studies should be further designed to investigate the effects of combined drug administration and to clarify the protective and harmful effects of salidroside on an organism. It is believed that with the further development of cytology and molecular biology, the application prospect of salidroside will be broader.

## Figures and Tables

**Table 1:** The content of salidroside.

No.	Sample	Production Place	Assay Method	Content
1	*R. rosea* L.	Xinjiang, Hebei, Russia	HPLC	0.34–0.48% [12]
2	*R. crenulata* (HK. f. et Thoms.) H. Ohba	Sichuan	HPLC	0.38–1.56% [15]
3	*R. Sachalinesis* Boriss	Changbai mountain	HPLC	1.09% [12]
4	*R. Kirilowii (Regel)* Maxim	Sichuan	HPLC	0.50% [12]
		Qinghai	HPLC	2.51% [13]
		Taibai mountain	HPLC	0.32% [22]
5	*R.sacra*	Tibet	HPLC	0.14% [12]
6	*R. henryi*	Sichuan	HPLC	0.66% [12]
7	*R. fastigiata*	Sichuan	HPLC	0.02% [12]
		Tibet	HPLC	0.53–0.83% [14]
8	*R. yunnanensis*	Yunnan	HPLC	0.30% [12]
9	*R. alsia*	Sichuan	HPLC	0.87% [12]
10	*R. wallichiana va R. cholaensis*	Sichuan	HPLC	0.02% [12]
11	*R. linearifolia*	Xinjiang	HPLC	1.64% [12]
12	*R. quadrifida*	Xinjiang	HPLC	1.13% [12]
		Qinghai	HPLC	2.12% [13]
13	*R. domulasa*	Beijing	HPLC	0.02% [12]
		Taibai mountain	HPLC	0 [22]
14	*R. tangutica*	Gansu	GC-MS	1.13% [24]
15	*R. taohoensis*	Gansu	HPLC	0 [22]
16	*R. crenulata*	Xizang	HPLC	0.74–1.32% [14]
17	*R. bupleuroides*	Xizang	HPLC	0.48% [14]
18	*R. rosea*	Xinjiang	HPLC	0.45–1.29% [19]
19	*R. algida*	Qinghai	HPLC	3.13% [13]
20	*R. coccinea*	Qinghai	HPLC	0.98% [13]
21	*R. juparensis*	Qinghai	HPLC	0.64% [13]
22	*R. Sachalinensis* A. Bor	Changbai mountain	HPLC	0.23–0.86% [20]
23	*R. wallichiana* var. *cholaensis*	Jilin Tonghua	HPLC	0.02–0.11% [21]
24	*R. eurycarpa*	Taibai mountain	HPLC	0.14% [22]
25	*R. henryi*	Taibai mountain	HPLC	0.04% [22]
26	*R. atropurpurea*	Sichuan	TLC	1.20–1.50 [23]

**Table 2:** Drugs of salidroside.

No.	Approval Number	Product Name	Composition	Production Manufacturers	Product Picture
1	Z20060362	Sofren Injection	*R.wallichiana* var. *cholaensis*	Tonghua Yusheng Pharmaceutical Co., Ltd.	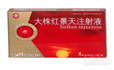
2	B20020080	Compound Alpine *Rhodiola* Oral Liquid	*R. sachalinensis* A. Bor, Astragalus and wolfberry	Shuzhong Pharmaceutical (Jilin) Co., Ltd.	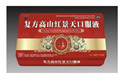
3	B20070002	*Rhodiola* Oral Liquid	*R. Crenulata* (HK. f. et Thoms.) H. Ohba	Tibet Tibetan Medicine Group Co., Ltd.	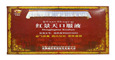
4	B20050055	Alpine *Rhodiola* Oral Liquid	*R. sachalinensis* A. Bor, auxiliary materials: sucrose, potassium sorbate	Hangzhou Huawei Pharmaceutical Co., Ltd.	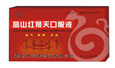
5	B20020480	Compound Rose *Rhodiola* Oral Liquid	*R. rosea* L., Szechuan lovage rhizome	Xinjiang Hua Shidan Pharmaceutical Co., Ltd.	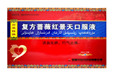
6	B20050013	*Rhodiola kirilowii (Regel) Maxim*. Tablet	*R. kirilowii (Regel) Maxim*.	Sichuan Elibis Pharmaceutical Co., Ltd.	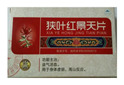
7	Z20050436	Large Plant *Rhodiola* Tablet	*R. wallichiana* var. *cholaensis*, total saponins of ginseng stems and leaves	Jiangsu Kangyuan Pharmaceutical Co., Ltd.	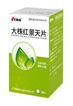
8	Z20040023	Dazhu *Rhodiola* Capsule	*R. wallichiana* var. *cholaensis*	Jiangsu Kangyuan Pharmaceutical Co., Ltd.	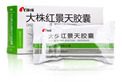

**Table 3:** Health products of salidroside.

No.	Approval Number	Product Name	Composition	Production Manufacturers	Product Picture
1	G20100243	Tongrentang Brand *Rhodiola* Capsule	*Rhodiola*	Beijing Tongrentang Health Pharmaceutical Co., Ltd.	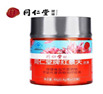
2	G20080605	Changxing Brand *Rhodiola* Soft Capsule	*Rhodiola* extracts	Guangdong Changxing Biotechnology Co., Ltd.	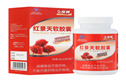
3	G20070151	Runtian Brand *Rhodiola* Pu‘er Tea	Pu‘er tea, *Rhodiola* extracts, pine pollen	Yunnan Blue Diamond Biotechnology Co., Ltd.	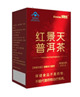
4	G20140664	Zhongyi Brand *Panax notoginseng* *Salvia miltiorrhiza Rhodiola* Oral Liquid	*Salvia miltiorrhiza*, *Panax notoginseng*, *Rhodiola*, *Panax ginseng*	Beijing Jinbi National Institute of Traditional Chinese Medicine	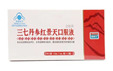
5	G20130763	Hehui Brand Kudzu Root *Codonopsis pilosula* (Franch.) Nannf. *Rhodiola* Granules	Kudzu root extracts, *Codonopsis pilosula* (Franch.) Nannf. extracts, *Hovenia dulcis* Thunb. extracts, *Rhodiola* extracts	Beijing Century Hehui Pharmaceutical Technology Co., Ltd.	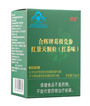
6	G20100246	Baibang Brand *Rhodiola* *Acanthopanax senticosus* *Ganoderma* Chewable Tablets	Ciwujia extracts, *Ganoderma* extracts, *Rhodiola* extracts	Shaanxi hundred years health Pharmaceutical Co., Ltd.	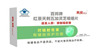
7	-	Saint Lotus Brand *Rhodiola* Tonic Wine	White wine, *R. sachalinensis* A. Bor, rock sugar	Beijing Shenglian Institute of Medicine and health products	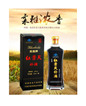
8	G20130020	Yuzhilin Brand *Rhodiola* Powder	*Rhodiola* extracts	Hebei Yuzhilin Pharmaceutical Co., Ltd.	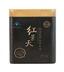
9	-	Adreset	*Rhodiola* extracts	Kamblex	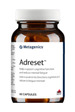
10	-	Rhodiola Crenul Ata Capsules	*Rhodiola* extracts	Kamblex	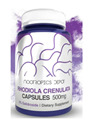
11	SC11344011106749	*Rhodiola* rosea Capsule	*Rhodiola* extracts	OME	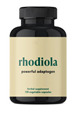
12	SC11344011402465	*Rhodiola* rosea Capsule	*Rhodiola* extracts	Shgrace	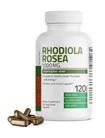

## Data Availability

Data are contained within the article.
